# Supplementary material for: The effect of environmental information on evolution of cooperation in stochastic games
Source: Nat Commun. 2023 Jul 12;14:4153. doi: 10.1038/s41467-023-39625-9 (PMC10338504; doi:10.1038/s41467-023-39625-9)
Supplement: Supplementary file 1 — Supplementary Information [file 41467_2023_39625_MOESM1_ESM.pdf]

Supplementary information:

The effect of environmental information on evolution of cooperation in stochastic games

Maria Kleshnina[1,*], Christian Hilbe[2,*], Štěpán Šimsa[3,4], Krishnendu Chatterjee[3], Martin A. Nowak[5]

[1] Institute for Advanced Study in Toulouse, Toulouse, France,
[2] Max Planck Institute for Evolutionary Biology, Plön, Germany,
[3] IST Austria, Klosterneuburg, Austria,
[4] Faculty of Mathematics and Physics, Charles University, Prague, Czech Republic,
[5] Department of Mathematics, Harvard University, Cambridge, USA,
Department of Organismic and Evolutionary Biology, Harvard University, Cambridge, USA

[*] These authors contributed equally to this work.

Contents

Supplementary Note 1: Games with deterministic transitions and weak selection **2**

Supplementary Note 2: Single-stochastic games **5**

Supplementary Note 3: A detailed analysis of some main text examples **6**
3.1 The timeout game . . . 6
3.2 The timeout game with conditional return . . . 11
3.3 On other deterministic transitions with a benefit of ignorance . . . 12
3.4 A single-stochastic game with conditional return . . . 12

Supplementary Note 4: Proofs and mathematical derivations **14**
4.1 The effect of game transformations and strategy transformations . . . 14
4.2 Analytical results for the transition vector $\mathbf{q} = (1, 0, 0; q, 0, 0)$ . . . 19

Supplementary References **22**

Supplementary Figures **23**

Supplementary Note 1: Games with deterministic transitions and weak selection

In this section, we summarize our results in the weak selection limit for games with deterministic transitions. Exact statements and all proofs are in Supplementary Note 4. To start with, consider a deterministic transition vector $\mathbf{q}=(q^1_{CC}, q^1_{CD}, q^1_{DD}, q^2_{CC}, q^2_{CD}, q^2_{DD})\in\{0,1\}^6$. In the limit of vanishing selection $\beta=0$, we can exploit some symmetry properties of the system, because payoffs become irrelevant for the evolutionary process. To describe these symmetries formally, it is useful to introduce some notation. For each stochastic game $\mathbf{q}$, we can define an associated *twin* $\chi_q(\mathbf{q})$ by relabelling the states,

$$\chi_q(\mathbf{q}) = (1-q^2_{CC}, 1-q^2_{CD}, 1-q^2_{DD},\; 1-q^1_{CC}, 1-q^1_{CD}, 1-q^1_{DD}). \tag{1}$$

Similarly, we can define an associated *mirror* game $\psi_q(\mathbf{q})$ by flipping the meaning of C and D,

$$\psi_q(\mathbf{q}) = (q^1_{DD}, q^1_{CD}, q^1_{CC}, q^2_{DD}, q^2_{CD}, q^2_{CC}). \tag{2}$$

We can also consecutively perform both transformations, yielding a *mirror-twin*,

$$\chi_q\circ\psi_q(\mathbf{q}) = (1-q^2_{DD}, 1-q^2_{CD}, 1-q^2_{CC},\; 1-q^1_{DD}, 1-q^1_{CD}, 1-q^1_{CC}). \tag{3}$$

We define analogous transformations for the players' memory-one strategies,

$$\begin{aligned}\chi_p(\mathbf{p}) &= (p^2_{CC}, p^2_{CD}, p^2_{DC}, p^2_{DD}, p^1_{CC}, p^1_{CD}, p^1_{DC}, p^1_{DD})\\ \psi_p(\mathbf{p}) &= (1-p^1_{DD}, 1-p^1_{DC}, 1-p^1_{CD}, 1-p^1_{CC}, 1-p^2_{DD}, 1-p^2_{DC}, 1-p^2_{CD}, 1-p^2_{CC})\end{aligned} \tag{4}$$

and for the invariant distributions,

$$\chi_v(\mathbf{v}) = (v^2_{CC}, v^2_{CD}, v^2_{DC}, v^2_{DD}, v^1_{CC}, v^1_{CD}, v^1_{DC}, v^1_{DD}), \tag{5}$$

$$\psi_v(\mathbf{v}) = (v^1_{DD}, v^1_{DC}, v^1_{CD}, v^1_{CC}, v^2_{DD}, v^2_{DC}, v^2_{CD}, v^2_{CC}). \tag{6}$$

With this notation, we can formulate a few useful relationships between a game with transition vector $\mathbf{q}$ and its associated twin, mirror, and mirror-twin. To this end, let $\mathbf{v}(\mathbf{p}|\mathbf{q})$ denote the stationary distribution among two players with strategy $\mathbf{p}$ interacting in the stochastic game with transition vector $\mathbf{q}$. We show the following relations (Supplementary Note 4, Lemma 1),

$$\mathbf{v}(\mathbf{p}|\mathbf{q})=\chi_v\Big(\,\mathbf{v}\big(\chi_p(\mathbf{p})\big|\chi_q(\mathbf{q})\big)\,\Big), \tag{7}$$

$$\mathbf{v}(\mathbf{p}|\mathbf{q})=\psi_v\Big(\,\mathbf{v}\big(\psi_p(\mathbf{p})\big|\psi_q(\mathbf{q})\big)\,\Big). \tag{8}$$

That is, suppose we know the invariant distribution $\mathbf{v}(\mathbf{p}|\mathbf{q})$ of the game $\mathbf{q}$ with respect to strategy $\mathbf{p}$.

Then we can directly infer the invariant distribution of the respective twin interaction – the one with transition vector $\chi_q(\mathbf{q})$ and strategies $\chi_p(\mathbf{p})$. Similarly, we can directly infer the invariant distribution of the respective mirror interaction, with $\psi_q(\mathbf{q})$ and $\psi_p(\mathbf{p})$. Now, if $\gamma(\mathbf{p}|\mathbf{q})$ denotes the average cooperation rate in a game with transition function $\mathbf{q}$ among two players with strategy $\mathbf{p}$, we obtain as a consequence (Supplementary Note 4, Corollary 1),

$$\gamma(\mathbf{p}|\mathbf{q}) = \gamma\big(\,\chi_p(\mathbf{p})\big|\chi_q(\mathbf{q})\,\big), \tag{9}$$

$$\gamma(\mathbf{p}|\mathbf{q}) = 1-\gamma\big(\,\psi_p(\mathbf{p})\,\big|\,\psi_q(\mathbf{q})\,\big). \tag{10}$$

This result depends on the particular strategy $\mathbf{p}$ used by the two players. However, as β becomes vanishingly small, we can derive an analogous statement independent of $\mathbf{p}$. To this end, for a given $\mathbf{q}$, let $\hat{\gamma}_0^F(\mathbf{q})$ be the average cooperation rate according to the invariant distribution of the full information game when individuals use deterministic strategies, in the limit of rare errors and $\beta = 0$. Then we show (Supplementary Note 4, Proposition 1) that

$$\hat{\gamma}_0^F(\mathbf{q}) = \hat{\gamma}_0^F\big(\chi_q(\mathbf{q})\big), \tag{11}$$

$$\hat{\gamma}_0^F(\mathbf{q}) = 1-\hat{\gamma}_0^F\big(\psi_q(\mathbf{q})\big). \tag{12}$$

There are two reasons why the relationships in (11) are useful. First, with each stochastic game $\mathbf{q}$ that we understand, we immediately understand three other stochastic games, $\chi_q(\mathbf{q})$, $\psi_q(\mathbf{q})$, and $\chi_q \circ \psi_q(\mathbf{q})$. This means that there are fewer distinct cases that need to be analyzed.

Second, in the special case that a transition vector $\mathbf{q}$ is its own mirror, $\psi_q(\mathbf{q}) = \mathbf{q}$, it follows directly from the second equation in (11) that $\hat{\gamma}_0^F(\mathbf{q}) = 1/2$. As we prove in Proposition 2 in Supplementary Note 4, in the no-information setting the respective average cooperation rates always satisfy $\hat{\gamma}_0^N(\mathbf{q}) = 1/2$, for all $\mathbf{q}$. The two results imply that for games with $\psi_q(\mathbf{q}) = \mathbf{q}$, the value of information is $V_0(\mathbf{q}) = \hat{\gamma}_0^F(\mathbf{q}) - \hat{\gamma}_0^N(\mathbf{q}) = 0$. Similarly, if $\hat{\gamma}_0^F(\mathbf{q}) < 1/2$ for some transition vector $\mathbf{q}$, then its mirror necessarily has $\hat{\gamma}_0^F(\psi_q(\mathbf{q})) > 1/2$. It follows that for each case $\mathbf{q}$ with a benefit of information we immediately obtain another case $\psi_q(\mathbf{q})$ in which there is a benefit of ignorance (of the same magnitude).

In general, we find that among the 64 deterministic games, exactly half of them is neutral. All these cases fall within four possible categories (see Supplementary Note 4, Proposition 3):

(1) The transition vector has an absorbing state: $q_{ij}^1 = 1$ or $q_{ij}^2 = 0$ for all $i, j \in \{C, D\}$.

(2) The transition vector is its own mirror: $\psi_q(\mathbf{q}) = \mathbf{q}$.

(3) The transition vector is its own mirror-twin: $\chi_q \circ \psi_q(\mathbf{q}) = \mathbf{q}$.

(4) The transition vector is state-independent: $q_{ij}^1 = q_{ij}^2$ for all $i, j \in \{C, D\}$.

As described in the main text, we can also define a simple proxy variable X that can be used to characterize whether information is beneficial, detrimental, or neutral in the limit of weak selection,

$$X = \Big(\mathbb{1}_{q^1_{CC}=1} + \mathbb{1}_{q^2_{CC}=0}\Big) - \Big(\mathbb{1}_{q^1_{DD}=1} + \mathbb{1}_{q^2_{DD}=0}\Big). \tag{13}$$

The rule is as follows: If one of the above four conditions (1) - (4) is satisfied, the game $\mathbf{q}$ is neutral. Moreover, in most of these cases, we have $X = 0$ (the only exception occurs if the transition vector has an absorbing state, in which case $X = -1$ and $X = 1$ is also possible). Otherwise, if none of the conditions (1) - (4) are satisfied, there is a benefit of information if $X > 0$ and a benefit of ignorance if $X < 0$. We illustrate these relationships in Fig. 4**a**. In that figure, blue bars indicate a benefit of information, and red bars indicate a benefit of ignorance. Moreover, Fig. 4**b,c** shows how the respective case numbers change as we vary the benefit of cooperation b_1 in state 1, and as we vary the strength of selection β.

Supplementary Note 2: Single-stochastic games

As described in the main text, a game is called single-stochastic if all entries in $\mathbf{q}$ but one are either zero or one (we refer to the remaining entry as q). It follows that there are $6 \cdot 2^5 = 192$ families of single-stochastic games. Out of those, we find that there are 24 transition vectors that have an absorbing state. By the same argument as before, information is neutral for those games, $V_\beta(\mathbf{q}) = 0$ for all β and q (see Proposition 3 in Supplementary Note 4). The other games can be analyzed numerically. Fig. S7–Fig. S10 show the respective results for no, weak, intermediate, and strong selection, respectively. In addition, Fig. S11 provides an overview of our numerical results across all $q \in [0, 1]$ and $\beta \in [10^{-3}, 10^{1}]$.

For the limit of no selection we find that the proxy variable X defined by (13) continues to make correct predictions in most cases. However, for 24 single-stochastic games we obtain $X = 0$ although the game does not exhibit neutral behavior for all $q \in [0, 1]$. Given the vector transformations (1)-(3), we only need to understand the behavior of 12 unique cases, where the stochastic transition q occurs only in state 1 of the game. We group these cases based on the transitions in state 2 (color names refer to Fig. S7),

(1) $\mathbf{q}_3 = (q00; 010)$ [yellow] and $\mathbf{q}_{19} = (q10; 010)$ [yellow];

(2) $\mathbf{q}_6 = (q00; 101)$ [blue] and $\mathbf{q}_{22} = (q10; 101)$ [blue];

(3) $\mathbf{q}_8 = (q00; 111)$ [blue] and $\mathbf{q}_{24} = (q10; 111)$ [blue];

(4) $\mathbf{q}_{10} = (q01; 001)$ [blue], $\mathbf{q}_{26} = (q11; 001)$ [blue], and $\mathbf{q}_{42} = (0q1; 001)$ [yellow];

(5) $\mathbf{q}_{12} = (q01; 011)$ [yellow], $\mathbf{q}_{28} = (q11; 011)$ [red], and $\mathbf{q}_{44} = (0q1; 011)$ [blue].

These cases can be classified into three different qualitative classes. (*i*) The first class includes the five cases $\mathbf{q}_6$, $\mathbf{q}_8$, $\mathbf{q}_{10}$, $\mathbf{q}_{22}$, $\mathbf{q}_{24}$. In all these cases, the respective game is neutral for $q = 0$, shows a benefit of information for $q = 1$, and also shows a benefit of information for all intermediate q values. (*ii*) The second class includes the four cases $\mathbf{q}_{26}$, $\mathbf{q}_{28}$, $\mathbf{q}_{42}$,$\mathbf{q}_{44}$. In these cases, the stochastic game is neutral for both $q = 0$ and $q = 1$, but for intermediate q they either exhibit a benefit of information, a benefit of ignorance, or both. (*iii*) Finally, the last class includes the three cases $\mathbf{q}_3$, $\mathbf{q}_{12}$, $\mathbf{q}_{19}$. In these cases, the stochastic game is neutral for $q = 0$, shows a benefit of information for $q = 1$, but shows a benefit of ignorance for some intermediate values of q.

Supplementary Note 3: A detailed analysis of some main text examples

After having provided some general information for games with deterministic or single-stochastic transitions, in the following we describe in more detail some of the specific examples that we have considered in the main text. In particular, we first discuss two the timeout game (Fig. 2**a–d**,Fig. 3**a,b**) and the timeout game with conditional return (Fig. 2**e–h**,Fig. 3**c,d**). In addition, we briefly discuss games with deterministic transitions that show a benefit of ignorance even for strong selection. Finally, we analyze the single-stochastic game highlighted in Fig. 5.

3.1 The timeout game

The first example we consider is the deterministic game with transition structure $\mathbf{q} = (1, 0, 0; 1, 1, 0)$. In the absence of selection, this game has a cooperation rate of $1/2$ for both games with and without information. However, increasing selection results in higher cooperation rates in the full-information setting. In order to understand why cooperation rates are different in games with and without information, we explore the stability of the strategies that are most abundant in each case.

Stability of $\mathbf{p} = (1, 0, 0, 0; x, 0, 0, 1)$.

In the game with full information, for strong selection and parameter values as in Fig. 2, the strategy $\mathbf{p} = (1, 0, 0, 0; x, 0, 0, 1)$ is most successful (Fig. 2**c**), with $x \in \{0, 1\}$. This strategy can therefore be implemented in two ways: either as *Grim-WSLS* $\mathbf{p} = (1, 0, 0, 0; 1, 0, 0, 1)$, or as *Grim-Risker* $\mathbf{p} = (1, 0, 0, 0; 0, 0, 0, 1)$ analyzed in Ref. 1. To explore if this strategy is a subgame perfect Nash equilibrium, we employ the one-shot deviation principle[2]. For this, we calculate continuation payoffs for each of the cases when players either use this strategy or deviate in one round and then return to using this strategy again. For this analysis, we assume that future payoffs may be discounted by a factor δ.

Let us first consider payoffs of non-deviating players. Given the nature of memory-1 strategies and state dependency of the game, we need to consider six possible cases, depending on the players' possible actions and environmental state.

1. CC in state 1. Then, the game transitions to state 1 and both players cooperate in all subsequent rounds, that is,
$$\pi_{CC,S1} = b_1 - c. \tag{14}$$
2. CD/DC in state 1. Then, the game transitions to state 2, where both players will defect. After that, the game returns to state 1 and both players still defect. Thereafter, the players return to state 2 in which they both cooperate. After this, the game returns to state 1, and players cooperate in all subsequent rounds. Therefore, the continuation payoff is given by
$$\pi_{CD,S1} = \delta^2(1-\delta)(b_2 - c) + \delta^3(b_1 - c). \tag{15}$$

3. DD in state 1. Here, the game transitions to state 2, where both players cooperate and, after returning to state 1, they cooperate in all subsequent rounds, that is,

$$\pi_{DD,S1} = (1-\delta)(b_2 - c) + \delta(b_1 - c). \tag{16}$$

4. CC in state 2. This case is similar to CC in state 1 since the game transitions to state 1 and both players cooperate in all subsequent rounds, that is,

$$\pi_{CC,S2} = b_1 - c. \tag{17}$$

5. CD/DC in state 2. Then, the game transitions to state 1, where both players defect. After, the game returns to state 2 and both players cooperate, which recovers cooperation in all subsequent rounds as the game returns to state 1. Then, the payoff is given by

$$\pi_{CD,S2} = \delta(1-\delta)(b_2 - c) + \delta^2(b_1 - c). \tag{18}$$

6. DD in state 2. Here, the game transitions to state 1, where both players defect, and, via cooperating in state 2, as before, players recover mutual cooperation in all subsequent rounds, that is,

$$\pi_{DD,S2} = \delta(1-\delta)(b_2 - c) + \delta^2(b_1 - c). \tag{19}$$

Now, let us derive the payoffs of the deviating players.

1. CC in state 1. In this case a deviating player defects for one round and then returns to the original strategy. After the mutant's defection, the game transitions to state 2 where both players defect. After the following mutual defection in state 1, players recover cooperation by cooperating in state 2 and cooperate in all subsequent rounds in state 1. That is,

$$\tilde{\pi}_{CC,S1} = (1-\delta)b_1 + \delta^3(1-\delta)(b_2 - c) + \delta^4(b_1 - c). \tag{20}$$

2. CD/DC in state 1. Here, the deviation requires the mutant to cooperate in state 2. After transitioning to state 1, both players defect, which again recovers mutual cooperation through transitioning to state 2. The payoff is then given by

$$\tilde{\pi}_{CD,S1} = -(1-\delta)c + \delta^2(1-\delta)(b_2 - c) + \delta^3(b_1 - c). \tag{21}$$

3. DD in state 1. As the game transitions to state 2, it requires that mutant defects while the second player cooperates. They still recover cooperation after mutual defection in state 1 as before. The

payoff of a deviating player is given by

$$\tilde{\pi}_{DD,S1} = (1-\delta)b_2 + \delta^2(1-\delta)(b_2 - c) + \delta^3(b_1 - c). \tag{22}$$

4. CC in state 2. This case is similar to CC in state 1 as yields the same payoff to the deviating player

$$\tilde{\pi}_{CC,S2} = (1-\delta)b_1 + \delta^3(1-\delta)(b_2 - c) + \delta^4(b_1 - c). \tag{23}$$

5. CD/DC in state 2. The deviating player will cooperate as game transitions to state 1 and the second player defects. This leads to the mutual defection in state 2 and a subsequent state 1. After this, both players cooperate in state 2 and all subsequent rounds in state 1. The payoff for this case is given by

$$\tilde{\pi}_{CD,S2} = -(1-\delta)c + \delta^3(1-\delta)(b_2 - c) + \delta^4(b_1 - c). \tag{24}$$

6. DD in state 2. Here, the game transitions to state 1, where the deviating player is required to cooperate. They again recover mutual cooperation as in the previous case after two rounds of mutual defection. The payoff is then given by

$$\tilde{\pi}_{DD,S2} = -(1-\delta)c + \delta^3(1-\delta)(b_2 - c) + \delta^4(b_1 - c). \tag{25}$$

In order for this strategy to be a subgame perfect Nash equilibrium, we need to check if it always yields higher payoffs than a one-shot deviation. For this, we require that

$$\pi_{CC,S1} \geq \tilde{\pi}_{CC,S1} \quad \text{and} \quad \pi_{CC,S2} \geq \tilde{\pi}_{CC,S2} \tag{26}$$

$$\pi_{CD,S1} \geq \tilde{\pi}_{CD,S1} \quad \text{and} \quad \pi_{CD,S2} \geq \tilde{\pi}_{CD,S2} \tag{27}$$

$$\pi_{DD,S1} \geq \tilde{\pi}_{DD,S1} \quad \text{and} \quad \pi_{DD,S2} \geq \tilde{\pi}_{DD,S2} \tag{28}$$

The respective inequalities for the payoffs after CD are always satisfied. The same is true for the inequality $\pi_{DD,S2} \geq \tilde{\pi}_{DD,S2}$. As payoffs in both states after mutual cooperation are identical, we only need to consider two cases.

1. We require $\pi_{CC} \geq \tilde{\pi}_{CC}$, which reduces to the following condition

$$(1+\delta+\delta^2)c \leq \delta(1+\delta+\delta^2)b_1 - \delta^3 b_2. \tag{29}$$

   For $\delta \to 1$ this inequality simplifies to $3c \leq 3b_1 - b_2$, which is satisfied for the parameters in Fig. 2.

2. We require $\pi_{DD,S1} \geq \tilde{\pi}_{DD,S1}$, which can be written as

$$(1+\delta)c \leq \delta(1+\delta)b_1 - \delta^2 b_2. \tag{30}$$

For $\delta \to 1$ this condition simplifies to $2c \leq 2b_1 - b_2$, which is also satisfied for the set of parameters we chose.

Stability of $WSLS$, $\mathbf{p} = (1,0,0,1;1,0,0,1)$.

Let us now apply a similar line of argument to $WSLS$. As can be seen in Fig. 2**c**, this strategy does neither evolve in the full-information nor in the no-information setting. First, we construct the payoffs for the non-deviating players.

1. CC in state 1. All players cooperate in all rounds, that is,

$$\pi_{CC,S1} = b_1 - c. \tag{31}$$

2. CD/DC in state 1. Then, players defect in state 2 and recover cooperation in all subsequent rounds in state 1, that is,

$$\pi_{CD,S1} = \delta(b_1 - c). \tag{32}$$

3. DD in state 1. The game transitions to state 2, where players cooperate, and then they cooperate in all subsequent rounds in state 1,

$$\pi_{DD,S1} = (1-\delta)(b_2 - c) + \delta(b_1 - c). \tag{33}$$

4. CC in state 2. Same as for CC in state 1,

$$\pi_{CC,S2} = b_1 - c. \tag{34}$$

5. CD/DC in state 2. Then, players defect in state 1 and recover cooperation in state 2 and cooperate in all subsequent rounds in state 1, that is,

$$\pi_{CD,S2} = \delta(1-\delta)(b_2 - c) + \delta^2(b_1 - c). \tag{35}$$

6. DD in state 2. The game transitions to state 1, where players cooperate in all subsequent rounds.

$$\pi_{DD,S2} = b_1 - c. \tag{36}$$

Payoffs of the deviating player then can be calculated in the following way.

1. CC in state 1. The deviating player defects in state 1 and after mutual defection in state 2, players recover mutual cooperation in state 1,

$$\tilde{\pi}_{CC,S1} = (1-\delta)b_1 + \delta^2(b_1 - c). \tag{37}$$

2. CD/DC in state 1. The deviating player cooperates in state 2, while the second player defects. This leads to mutual defection in state 1, followed by mutual cooperation in state 2 and subsequent cooperation in all rounds in state 1,

$$\tilde{\pi}_{CD,S1} = -(1-\delta)c + \delta^2(1-\delta)(b_2 - c) + \delta^3(b_1 - c). \tag{38}$$

3. DD in state 1. The deviating player defects in state 2, which leads to mutual defection in state 1 and recovery of mutual cooperation via state 2,

$$\tilde{\pi}_{DD,S1} = (1-\delta)b_2 + \delta^2(1-\delta)(b_2 - c) + \delta^3(b_1 - c). \tag{39}$$

4. CC in state 2. Same as for CC in state 1,

$$\tilde{\pi}_{CC,S2} = (1-\delta)b_1 + \delta^2(b_1 - c). \tag{40}$$

5. CD/DC in state 2. The deviating player cooperates in state 1, while the second player defects. This leads to mutual defection in state 2, followed by mutual cooperation in all rounds in state 1,

$$\tilde{\pi}_{CD,S2} = -(1-\delta)c + \delta^2(b_1 - c). \tag{41}$$

6. DD in state 2. The deviating player defects in state 1, which leads to mutual defection in state 2 and recovery of mutual cooperation,

$$\tilde{\pi}_{DD,S2} = (1-\delta)b_1 + \delta^2(b_1 - c). \tag{42}$$

As before, in each case we compare the payoffs for deviating and non-deviating players. We see that non-deviating players are better off whenever different actions were played in the previous round independent of the state. Conditions $\pi_{CC} \geq \tilde{\pi}_{CC}$, $\pi_{DD,S1} \geq \tilde{\pi}_{DD,S1}$ and $\pi_{DD,S2} \geq \tilde{\pi}_{DD,S2}$ simplify to

$$(1+\delta)c \leq \delta b_1, \tag{43}$$

$$(1+\delta)c \leq \delta(1+\delta)b_1 - \delta^2 b_2 \tag{44}$$

While the second condition can be rewritten as $2c \leq 2b_1 - b_2$ for $\delta \to 1$ and is satisfied, the first condition challenges the stability of $WSLS$. As δ approaches 1, this condition can be written as $2c \leq b_1$. For our set of parameters, this condition is not satisfied, which explains why we rarely observe players adopting this strategy.

In order to analyze strategies for the game without information, we can consider two cases. First, when players can deduct the current state based on the previous round outcome (assuming they can take into account payoffs they achieve and the actions) but cannot condition their strategy on the current state

as such and use this information only for computing the payoffs. This case will be similar in the analysis as the one-shot deviation principle considered for the game with full information. Since $WSLS$ is not an equilibrium strategy, it explains why we mostly observe players using $Grim$ and $ALLD$. Second, if we assume that players cannot take into account information about previous payoffs and, hence, cannot predict the current state, we analyse the ability of other strategies to invade a population of $WSLS$ players by comparing payoffs mutants can achieve. Note that it is sufficient to consider only strategies like $Grim$, $ALLD$ and $Risker$. The corresponding payoffs $\pi(x, y)$ of a player adopting strategy x against a player adopting strategy y are then given by

$$\pi(WSLS, WSLS) = b_1 - c + \mathcal{O}(\epsilon) \tag{45}$$

$$\pi(Grim, WSLS) = \frac{1}{35}(15b_1 + 6b_2 - 7c) + \mathcal{O}(\epsilon) \tag{46}$$

$$\pi(ALLD, WSLS) = \frac{2}{7}b_1 + \frac{3}{14}b_2 + \mathcal{O}(\epsilon) \tag{47}$$

$$\pi(Risker, WSLS) = \frac{2}{3}b_1 - \frac{1}{3}c + \mathcal{O}(\epsilon). \tag{48}$$

In particular, $WSLS$ can only withstand an invasion by $Risker$ if $2c < b_1$.

3.2 The timeout game with conditional return

This game (deterministic case 38) has the transition vector $\mathbf{q} = (1, 0, 0; 1, 1, 0)$. Similarly to the timeout game, in the absence of selection this game has a cooperation rate of $1/2$ in both settings, with and without information. However, for larger selection strengths, these results can change. In particular for strong selection, we observe a considerable benefit of ignorance when b_1 is sufficiently large (Fig. 2**h**). To explain this observation, we again characterize the stability of the most abundant strategies.

For full information, there are three most abundant strategies for the parameter set we chose (Fig. 2**g**): $(1, 0, 0, x; y, 0, 0, 1)$, $(1, 0, 1, x; y, 0, 0, 1)$ and $(1, 1, 1, x; y, 0, 1, 0)$, where $x, y \in \{0, 1\}$. All three strategies are self-cooperating and cooperate among each other. However, according to a one-shot deviation analysis, only the first strategy $(1, 0, 0, x; y, 0, 0, 1)$ is subgame perfect. One key difference from the game with transition vector $\mathbf{q}_{39} = (1, 0, 0;\ 1, 1, 1)$ that makes $WSLS$ a subgame perfect Nash equilibrium is that mutual defection in state 2 leads to the game remaining in the worse state 2. Then, deviations from $WSLS$ necessarily yield lower payoff than the payoff of non-deviating players.

Without information, the payoffs that residents and mutants achieve for the most common non-self-cooperating strategies are given by

$$\pi(WSLS, WSLS) = b_1 - c + \mathcal{O}(\epsilon) \tag{49}$$

$$\pi(Grim, WSLS) = \frac{1}{5}(b_1 + 2b_2 - c) + \mathcal{O}(\epsilon) \tag{50}$$

$$\pi(ALLD, WSLS) = \frac{b_2}{2} + \mathcal{O}(\epsilon) \tag{51}$$

$$\pi(Risker, WSLS) = \frac{1}{3}(b_1 + b_2 - c) + \mathcal{O}(\epsilon) \tag{52}$$

A population of $WSLS$ players is stable against any of these other strategies if $b_2 < 2(b_1 - c)$.

3.3 On other deterministic transitions with a benefit of ignorance

In Table S1 we summarize all games that show some benefit of ignorance for sufficiently large selection strength β and sufficiently large benefit b_1 in state 1.

Game number	Transition vector	Maximum value of the benefit of ignorance
38	$(100; 110)$	0.0904
44	$(101; 100)$	0.0006
46	$(101; 110)$	0.1288
52	$(110; 100)$	0.0014
53	$(110; 101)$	0.0188
55	$(110; 111)$	0.0191

Table S1: Games with some benefit of ignorance

The table suggests that games with a benefit of ignorance are rare. Moreover, for only two games this benefit is substantial, for game $\mathbf{q}_{38} = (100; 110)$ considered in Fig. 2**e–h** and game $\mathbf{q}_{46} = (101; 110)$. The transition function of game 46 differs from game 38 only in one entry (if both players defect in state 1, they remain in that state). As a general pattern, we observe that all transitions in Table S1 have the property that individuals move to state 1 if they both cooperated. Moreover, according to all of these games, some forms of defection in state 1 are punished with a transition to state 2. However, these two patterns cannot be used to fully characterize the games in Table S1; there are games that satisfy both properties without exhibiting a benefit of ignorance in the limit of strong selection (e.g. games 37 and 53 in Fig. S4). A consistent classification becomes even more complicated once we consider single-stochastic transition function (Fig. S7-Fig. S11).

3.4 A single-stochastic game with conditional return

Next, we consider the stochastic game considered in Fig. 5, with transition vector $\mathbf{q} = (1, 0, 0,\ q, 0, 0)$. According to this game, players find themselves in the less profitable state 2 if one or both players defected in the previous round. Otherwise, if both players cooperated, they remain in state 1 with certainty if they are already there, or they move towards state 1 with probability q if they start out in state 2.

Because the game transitions for sure to the second state after any defection, it follows that for strategies in the full-information setting the entries p^1_{CD}, p^1_{DC}, p^1_{DD} are irrelevant (players never are to make any decision in state 1 after some player defected previously). This implies that all strategies of the form $\mathbf{p} = (p^1_{CC}, x, y, z;\ p^2_{CC}, p^2_{CD}, p^2_{DC}, p^2_{DD})$ are behaviorally equivalent, for all $x, y, z \in [0, 1]$.

Hence, it suffices to consider a simplified 5-dimensional strategy space containing all strategies of the form $\mathbf{p}=(p^1_{CC};\ p^2_{CC}, p^2_{CD}, p^2_{DC}, p^2_{DD})$. These are the strategies that we depict in Fig. 5**h**.

The numerical simulations depicted in Fig. 5 show that there is a benefit of ignorance for a wide range of transition probabilities q and selection strengths β. We can further support these numerical results by considering the limits of weak and strong selection, respectively. In Supplementary Note 4, we derive the following two results for weak selection,

1. No selection (Propositions 2 and 4): For $\beta = 0$, the no-information setting yields an average cooperation rate of $\hat{\gamma}^N(\mathbf{q}) = 1/2$. In contrast, full information yields an average cooperation rate of $\hat{\gamma}^F(\mathbf{q}) = 1/2 - \frac{3q(1-q)}{64(1+q)}$. In particular, there is a benefit of ignorance $V_0(\mathbf{q}) < 0$ for all $q \in (0,1)$.

2. Weak selection: The above result generalizes to positive but sufficiently small selection strengths. That is, for any given $q \in (0,1)$ there is a threshold $\hat{\beta}_q$ such that for all $\beta < \hat{\beta}_q$ there is a benefit of ignorance $V_\beta(\mathbf{q}) < 0$.

To gain some intuition for dynamics under strong selection, we characterize the game's Nash equilibria among the pure memory-1 strategies, both for full information and no information. For no information, there are three Nash equilibria for the parameters used in Fig. 5. These equilibria are $ALLD{=}\,(0,0,0,0)$, $Grim{=}\,(1,0,0,0)$ and $WSLS{=}\,(1,0,0,1)$. Out of those, only $WSLS$ is able to sustain cooperation in the presence of rare errors – that is, only for $WSLS$ we have $\lim_{\varepsilon\to 0}\gamma(\mathbf{p},\mathbf{p}) = 1$. For full information, we find six distinct equilibria that correspond to

$$\begin{aligned} ALLD &= (0;0,0,0,0), & Grim &= (1;1,0,0,0),\\ ALLD\text{-}Grim &= (0;1,0,0,0), & Grim\text{-}ALLD &= (1;0,0,0,0),\\ WSLS &= (1;1,0,0,1), & AWSLS &= (1;0,0,0,1). \end{aligned}$$

Out of those, only $WSLS$ and $AWSLS$ sustain cooperation in the presence of rare errors.

Supplementary Note 4: Proofs and mathematical derivations

In the following, we provide the proofs of our analytical statements. To remind the reader of the meaning of our mathematical notations, we provide a summary in Table S2.

Symbol	Description
$\mathbf{p}$	memory-one strategy of a player
$\mathbf{q}$	environmental transition vector
$\mathcal{S}_N,\ \mathcal{S}_F$	set of all memory-1 strategies in the no-information and full-information setting
$\mathcal{P}_N,\ \mathcal{P}_F$	set of all deterministic memory-1 strategies
$s_i \in \{s_1, s_2\}$	environmental state
$b_1,\ b_2,\ c$	parameters of the game (benefits in state 1 and 2 and cost)
β	selection strength
ε	error rate
δ	continuation probability
$M(\mathbf{p}\vert\mathbf{q})$	transition matrix when two players with strategy $\mathbf{p}$ interact in game with vector $\mathbf{q}$
$\mathbf{v}(\mathbf{p}\vert\mathbf{q})$	stationary distribution of $M(\mathbf{p}\vert\mathbf{q})$
$\gamma(\mathbf{p}\vert\mathbf{q})$	Resulting average cooperation rate, given strategies $\mathbf{p}$ and transition vector $\mathbf{q}$
$\hat{\gamma}^N,\ \hat{\gamma}^F$	cooperation rates for populations with no information and with full information
$\pi(\mathbf{p},\mathbf{q})$	payoff of strategy $\mathbf{p}$ given transition vector $\mathbf{q}$
ρ	probability to switch to a different strategy
$V_\beta(\mathbf{q})$	value of information in a game $\mathbf{q}$
X	proxy-variable measuring the value of information
$\chi(\mathbf{q})$	twin transformation of $\mathbf{q}$, $\chi_q(\mathbf{q}) = (1-q^2_{CC}, 1-q^2_{CD}, 1-q^2_{DD},\ 1-q^1_{CC}, 1-q^1_{CD}, 1-q^1_{DD})$
$\psi(\mathbf{q})$	mirror transformation of $\mathbf{q}$, $\psi_q(\mathbf{q}) = (q^1_{DD}, q^1_{CD}, q^1_{CC}, q^2_{DD}, q^2_{CD}, q^2_{CC})$

Table S2: Table of notation

4.1 The effect of game transformations and strategy transformations

In the following, we derive the results summarized in Supplementary Note 1. We begin by introducing some additional notation. First, for a given memory-1 strategy $\mathbf{p}$ we write

$$\mathbf{p} = (p^1_{CC}, p^1_{CD}, p^1_{DC}, p^1_{DD}, p^2_{CC}, p^2_{CD}, p^2_{DC}, p^2_{DD}) = (\mathbf{p^1}, \mathbf{p}^2). \tag{53}$$

Similarly, and slightly abusing our notation, we can write a 6-dimensional transition vector $\mathbf{q}$ as an 8-dimensional vector

$$\mathbf{q} = (q^1_{CC}, q^1_{CD}, q^1_{DC}, q^1_{DD}, q^2_{CC}, q^2_{CD}, q^2_{DC}, q^2_{DD}) = (\mathbf{q^1}, \mathbf{q}^2), \tag{54}$$

with the restriction that transitions are required to be symmetric, $q^i_{CD} = q^i_{DC}$ for $i \in \{1, 2\}$. Now consider two players with strategy $\mathbf{p}$ who interact in a stochastic game with transition vector $\mathbf{q}$. Using the above

notation, we can write the transition matrix of the resulting Markov chain as

$$M(\mathbf{p}|\mathbf{q}) = \left(\begin{array}{c|c} \big((\mathbf{q}^1)^\intercal\,\mathbf{1}\big) \otimes A(\mathbf{p^1}) & \big((\mathbf{1}-\mathbf{q}^1)^\intercal\,\mathbf{1}\big) \otimes A(\mathbf{p^2}) \\ \hline \big((\mathbf{q}^2)^\intercal\,\mathbf{1}\big) \otimes A(\mathbf{p^1}) & \big((\mathbf{1}-\mathbf{q}^2)^\intercal\,\mathbf{1}\big) \otimes A(\mathbf{p^2}) \end{array}\right). \tag{55}$$

Here, $(\mathbf{q^i})^\intercal$ is the transpose of $\mathbf{q^i}$, $\mathbf{1}$ is a vector of ones, $\otimes$ is a Hadamard product (entry-wise multiplication), and

$$A(\mathbf{p^i}) = \begin{pmatrix} p^i_{CC}p^i_{CC} & p^i_{CC}(1-p^i_{CC}) & (1-p^i_{CC})p^i_{CC} & (1-p^i_{CC})(1-p^i_{CC}) \\ p^i_{CD}p^i_{DC} & p^i_{CD}(1-p^i_{DC}) & (1-p^i_{CD})p^i_{DC} & (1-p^i_{CD})(1-p^i_{DC}) \\ p^i_{DC}p^i_{CD} & p^i_{DC}(1-p^i_{CD}) & (1-p^i_{DC})p^i_{CD} & (1-p^i_{DC})(1-p^i_{CD}) \\ p^i_{DD}p^i_{DD} & p^i_{DD}(1-p^i_{DD}) & (1-p^i_{DD})p^i_{DD} & (1-p^i_{DD})(1-p^i_{DD}) \end{pmatrix} \tag{56}$$

Using this notation, we can prove the two relationships formulated in Eq. (7).

Lemma 1. *Consider an effective memory-one strategy* $\mathbf{p} \in \mathcal{S}_N$ *and a transition vector* $\mathbf{q}$ *such that the invariant distribution* $\mathbf{v}(\mathbf{p}|\mathbf{q})$ *is well-defined (i.e., the stationary distribution is unique). Then also* $\mathbf{v}\big(\,\psi_p(\mathbf{p})\big|\psi_q(\mathbf{q})\,\big)$ *and* $\mathbf{v}\big(\,\psi_p(\mathbf{p})\big|\psi_q(\mathbf{q})\,\big)$ *are well-defined. Moreover, the following relationships hold,*

(i) $\mathbf{v}(\mathbf{p}|\mathbf{q}) = \chi_v\big(\,\mathbf{v}\big(\chi_p(\mathbf{p})\big|\chi_q(\mathbf{q})\big)\,\big)$,

(ii) $\mathbf{v}(\mathbf{p}|\mathbf{q}) = \psi_v\big(\,\mathbf{v}\big(\psi_p(\mathbf{p})|\psi_q(\mathbf{q})\big)\,\big)$.

Proof. For the proof, we use permutation matrices.

(*i*) We note that if $M := M(\mathbf{p}|\mathbf{q})$ is the transition matrix with respect to the original vectors $\mathbf{p}$ and $\mathbf{q}$, then the transition matrix $M_\chi := M\big(\,\chi_p(\mathbf{p})\big|\chi_q(\mathbf{q})\big)$ satisfies

$$E_\chi M_\chi E_\chi = M. \tag{57}$$

In this identity, E_χ is the permutation matrix

$$E_\chi = \left(\begin{array}{c|c} \mathbf{0} & \tilde{E}_\chi \\ \hline \tilde{E}_\chi & \mathbf{0} \end{array}\right), \tag{58}$$

where

$$\tilde{E}_\chi = \begin{pmatrix} 1 & 0 & 0 & 0 \\ 0 & 1 & 0 & 0 \\ 0 & 0 & 1 & 0 \\ 0 & 0 & 0 & 1 \end{pmatrix}. \tag{59}$$

Now, if $\mathbf{v} := \mathbf{v}(\mathbf{p}, \mathbf{q})$ is a stationary distribution of M, we have

$$\mathbf{v} = \mathbf{v}M = \mathbf{v}E_\chi M_\chi E_\chi. \tag{60}$$

By multiplying E_χ from the right and noting that E_χ^2 is the identity matrix, we conclude

$$(\mathbf{v}E_\chi) = (\mathbf{v}E_\chi)M_\chi. \tag{61}$$

It follows that if $\mathbf{v}$ is the unique invariant distribution of M, then $\mathbf{v}E_\chi$ is the unique invariant distribution of M_χ. A straightforward calculation confirms that $\chi_v\big(\mathbf{v}E_\chi\big) = \mathbf{v}$.

(*ii*) The proof of the second identity is analogous; we only need to replace the permutation matrix by

$$E_\psi = \left(\begin{array}{c|c} \tilde{E}_\psi & \mathbf{0} \\ \hline \mathbf{0} & \tilde{E}_\psi \end{array}\right), \tag{62}$$

where

$$\tilde{E}_\psi = \begin{pmatrix} 0 & 0 & 0 & 1 \\ 0 & 0 & 1 & 0 \\ 0 & 1 & 0 & 0 \\ 1 & 0 & 0 & 0 \end{pmatrix}. \tag{63}$$

□

As an immediate consequence of the above lemma, we can also derive formulas for the respective average cooperation rates, as summarized in (9). The precise statement is as follows.

Corollary 1. *Consider an effective memory-one strategy* $\mathbf{p} \in \mathcal{S}_N$ *and a transition vector* $\mathbf{q}$ *such that the resulting average cooperation rate* $\gamma(\mathbf{p}|\mathbf{q})$ *is well-defined. Then*

$$\gamma(\mathbf{p}|\mathbf{q}) = \gamma\big(\,\chi_p(\mathbf{p})\big|\chi_q(\mathbf{q})\,\big) \quad \textit{and} \quad \gamma(\mathbf{p}|\mathbf{q}) = 1 - \gamma\big(\,\psi_p(\mathbf{p})|\psi_q(\mathbf{q})\,\big). \tag{64}$$

Proof. In the main text, we have defined the respective average cooperation rate as for $\mathbf{v} := \mathbf{v}(\mathbf{p}, \mathbf{q})$ as

$$\gamma(\mathbf{p}|\mathbf{q}) = v^1_{CC} + \frac{v^1_{CD} + v^1_{DC}}{2} + v^2_{CC} + \frac{v^2_{CD} + v^2_{DC}}{2}. \tag{65}$$

By Lemma 1(*i*), we have

$$\mathbf{v}\big(\,\chi_p(\mathbf{p})\big|\chi_q(\mathbf{q})\,\big) = (v^2_{CC}, v^2_{CD}, v^2_{DC}, v^2_{DD}, v^1_{CC}, v^1_{CD}, v^1_{DC}, v^1_{DD}). \tag{66}$$

Therefore,

$$\gamma\big(\,\chi_p(\mathbf{p})\big|\chi_q(\mathbf{q})\,\big) = v^2_{CC}+\frac{v^2_{CD}+v^2_{DC}}{2}+v^1_{CC}+\frac{v^1_{CD}+v^1_{DC}}{2} = \gamma(\mathbf{p}|\mathbf{q}). \tag{67}$$

Analogously, because of Lemma 1(*ii*), we have

$$\mathbf{v}\big(\,\psi_p(\mathbf{p})\big|\psi_q(\mathbf{q})\,\big) = (v^1_{DD}, v^1_{DC}, v^1_{CD}, v^1_{CC}, v^2_{DD}, v^2_{DC}, v^2_{CD}, v^2_{CC}). \tag{68}$$

It follows that

$$\begin{aligned}\gamma\big(\,\psi_p(\mathbf{p})\big|\psi_q(\mathbf{q})\,\big) &= v^1_{DD}+\frac{v^1_{DC}+v^1_{CD}}{2}+v^2_{DD}+\frac{v^2_{DC}+v^2_{CD}}{2}\\ &= 1-\left(v^1_{CC}+\frac{v^1_{CD}+v^1_{DC}}{2}+v^2_{CC}+\frac{v^2_{CD}+v^2_{DC}}{2}\right) = 1-\gamma(\mathbf{p}|\mathbf{q}).\end{aligned} \tag{69}$$

□

The above results hold for any given strategy $\mathbf{p}$ and transition vector $\mathbf{q}$ (provided that the invariant distribution of the resulting game dynamics is unique). In the limit of rare mutations and weak selection, we can use these results to compute the average cooperation rate in evolving populations (see also Eq. (11)),

Proposition 1. *Consider a stochastic game with transition vector* $\mathbf{q}\neq(1,1,1,0,0,0)$. *Moreover, suppose players have full information and they can choose among all deterministic memory-one strategies. Let* $\hat{\gamma}^F_0(\mathbf{q})$ *denote the average cooperation rate across time, if the population evolves according to a process with rare mutations and no selection (for an arbitrary error rate* $\varepsilon>0$*). Then*

$$\hat{\gamma}^F_0(\mathbf{q}) = \hat{\gamma}^F_0\big(\chi_q(\mathbf{q})\big) \quad \textit{and} \quad \hat{\gamma}^F_0(\mathbf{q}) = 1-\hat{\gamma}^F_0\big(\psi_q(\mathbf{q})\big). \tag{70}$$

In particular, the relationships remain true in the limit of rare errors $\varepsilon\to 0$.

Proof. Because we consider the limit of rare mutations and no selection, all possible resident strategies $\mathbf{p}\in\mathcal{P}_F$ are equally likely to be played. Because of Corollary 1, and because the map $\chi_p:\mathcal{P}_F\to\mathcal{P}_F$ is a bijection, we obtain

$$\hat{\gamma}^F_0(\mathbf{q}) = \sum_{\mathbf{p}\in\mathcal{P}_F}\frac{\gamma(\mathbf{p}|\mathbf{q})}{|\mathcal{P}_F|} = \sum_{\mathbf{p}\in\mathcal{P}_F}\frac{\gamma\big(\,\chi_p(\mathbf{p})\big|\chi_q(\mathbf{q})\,\big)}{|\mathcal{P}_F|} = \sum_{\mathbf{p}\in\mathcal{P}_F}\frac{\gamma\big(\,\mathbf{p}\big|\chi_q(\mathbf{q})\,\big)}{|\mathcal{P}_F|} = \hat{\gamma}^F_0\big(\chi_q(\mathbf{q})\big). \tag{71}$$

Similarly, we obtain

$$\hat{\gamma}^F_0(\mathbf{q}) = \sum_{\mathbf{p}\in\mathcal{P}_F}\frac{\gamma(\mathbf{p}|\mathbf{q})}{|\mathcal{P}_F|} = \sum_{\mathbf{p}\in\mathcal{P}_F}\frac{1-\gamma\big(\psi_p(\mathbf{p})\big|\psi_q(\mathbf{q})\big)}{|\mathcal{P}_F|} = \sum_{\mathbf{p}\in\mathcal{P}_F}\frac{1-\gamma\big(\mathbf{p}\big|\psi_q(\mathbf{q})\big)}{|\mathcal{P}_F|} = 1-\hat{\gamma}^F_0\big(\psi_q(\mathbf{q})\big). \tag{72}$$

□

In the no-information setup, the corresponding average cooperation rate takes an even simpler form, as the following result shows.

Proposition 2. *Consider a transition vector* $\mathbf{q} \neq (1,1,1,0,0,0)$ *and suppose players can choose among all deterministic memory-one no-information strategies. Let* $\hat{\gamma}_0^N(\mathbf{q})$ *denote the respective average cooperation rate under the evolutionary process with rare mutations and no selection (for an arbitrary error rate* ε*). Then* $\hat{\gamma}_0^N(\mathbf{q}) = 1/2$.

Proof. Because players do not condition their behavior on the environmental state, it follows that their average cooperation rate does not depend on the game's transition vector $\mathbf{q}$. In particular, for $\mathbf{p} \in \mathcal{P}_N$ and $\mathbf{q} \neq (1,1,1,0,0,0)$ we have

$$\gamma\big(\mathbf{p}|\psi_q(\mathbf{q})\big) = \gamma(\mathbf{p}|\mathbf{q}). \tag{73}$$

Because $\psi_p : \mathcal{P}_N \to \mathcal{P}_N$ is bijective and $\psi_p^2 = \text{Id}$, we can therefore use Corollary 1(*ii*) to conclude

$$\begin{aligned}
\hat{\gamma}_0^N(\mathbf{q}) &= \sum_{\mathbf{p}\in\mathcal{P}_N} \frac{\gamma(\mathbf{p}|\mathbf{q})}{|\mathcal{P}_N|} = \sum_{\mathbf{p}\in\mathcal{P}_N} \frac{\gamma(\mathbf{p}|\mathbf{q}) + \gamma(\mathbf{p}|\mathbf{q})}{2|\mathcal{P}_N|} = \sum_{\mathbf{p}\in\mathcal{P}_N} \frac{\gamma(\mathbf{p}|\mathbf{q}) + \gamma\big(\psi_p(\mathbf{p})|\mathbf{q}\big)}{2|\mathcal{P}_N|} \\
&= \sum_{\mathbf{p}\in\mathcal{P}_N} \frac{\gamma(\mathbf{p}|\mathbf{q}) + 1 - \gamma\big(\psi_p^2(\mathbf{p})|\psi_q(\mathbf{q})\big)}{2|\mathcal{P}_N|} = \sum_{\mathbf{p}\in\mathcal{P}_N} \frac{\gamma(\mathbf{p}|\mathbf{q}) + 1 - \gamma\big(\mathbf{p}|\psi_q(\mathbf{q})\big)}{2|\mathcal{P}_N|} \\
&= \sum_{\mathbf{p}\in\mathcal{P}_N} \frac{\gamma(\mathbf{p}|\mathbf{q}) + 1 - \gamma(\mathbf{p}|\mathbf{q})}{2|\mathcal{P}_N|} = \frac{1}{2}.
\end{aligned} \tag{74}$$

□

By combining Propositions 1 and 2, we can describe for which games information is neutral.

Proposition 3. *Consider a stochastic game with transition vector* $\mathbf{q} \neq (1,1,1,0,0,0)$ *and suppose one of the following four conditions is satisfied.*

(1) The vector has an absorbing state: $q_{ij}^1 = 1$ *or* $q_{ij}^2 = 0$ *for all* $i,j \in \{C,D\}$.

(2) The vector is its own mirror: $\psi_q(\mathbf{q}) = \mathbf{q}$.

(3) The vector is its own mirror-twin: $\chi_q \circ \psi_q(\mathbf{q}) = \mathbf{q}$.

(4) The vector is deterministic and state-independent, $q_{ij}^1 = q_{ij}^2 \in \{0,1\}$ *for all* $i,j \in \{C,D\}$.

Then, in the limiting case of rare mutations and no selection, information is neutral, $V_0(\mathbf{q}) = 0$. *Moreover, in case (1) and (4), the same result holds for positive selection strengths* $V_\beta(\mathbf{q}) = 0$ *for all* $\beta \geq 0$.

Proof. (1) Suppose without loss of generality that the unique absorbing state is state 1. Then, for any given full-information strategy $\mathbf{p} = (p_{CC}^1, p_{CD}^1, p_{DC}^1, p_{DD}^1, p_{CC}^2, p_{CD}^2, p_{DC}^2, p_{DD}^2) \in \mathcal{P}_F$, we define

a corresponding no-information strategy $\tilde{\mathbf{p}} \in \mathcal{P}_N$ by

$$\tilde{\mathbf{p}} = (p^1_{CC}, p^1_{CD}, p^1_{DC}, p^1_{DD}, p^1_{CC}, p^1_{CD}, p^1_{DC}, p^1_{DD}). \tag{75}$$

Because the first state is absorbing, eventually only the first half of the memory-1 strategy is relevant for a player's decision making. Therefore, $\gamma(\tilde{\mathbf{p}}|\mathbf{q}) = \gamma(\mathbf{p}|\mathbf{q})$ for all $\mathbf{p} \in \mathcal{P}_F$. Moreover, since each $\tilde{\mathbf{p}} \in \mathbf{p}$ has the same number of pre-images under this mapping, it follows that the average cooperation rate under full information coincides with the average cooperation rate under no information. We note that this argument does not require selection strength to be zero; it is merely based on the insight that for any full-information strategy there is a unique no-information strategy that gives rise to exactly the same behavior.

(2) If $\psi_q(\mathbf{q}) = \mathbf{q}$, it follows from Proposition 1 that

$$\hat{\gamma}_0^F(\mathbf{q}) = 1 - \hat{\gamma}_0^F\big(\psi_q(\mathbf{q})\big) = 1 - \hat{\gamma}_0^F(\mathbf{q}). \tag{76}$$

Therefore, $\hat{\gamma}_0^F(\mathbf{q}) = 1/2 = \hat{\gamma}_0^N(\mathbf{q})$.

(3) The case $\chi_q \circ \psi_q(\mathbf{q}) = \mathbf{q}$ follows analogously. Again by Proposition 1,

$$\hat{\gamma}_0^F(\mathbf{q}) = 1 - \hat{\gamma}_0^F\big(\psi_q(\mathbf{q})\big) = 1 - \hat{\gamma}_0^F\big(\chi_q \circ \psi_q(\mathbf{q})\big) = 1 - \hat{\gamma}_0^F(\mathbf{q}). \tag{77}$$

(4) The case of deterministic and state-independent transitions is similar to case (1). For each previous history $(a, \tilde{a}) \in \{C, D\}^2$, let $s_{a,\tilde{a}} \in \{s_1, s_2\}$ denote the unique environmental state that is reached after that history. Then, for each $\mathbf{p} \in \mathcal{P}_F$ we can define a behaviorally equivalent no-memory strategy $\tilde{\mathbf{p}} \in \mathcal{P}$ by

$$\tilde{\mathbf{p}} = (p^{s_{CC}}_{CC}, p^{s_{CD}}_{CD}, p^{s_{DC}}_{DC}, p^{s_{DD}}_{DD}, p^{s_{CC}}_{CC}, p^{s_{CD}}_{CD}, p^{s_{DC}}_{DC}, p^{s_{DD}}_{DD}). \tag{78}$$

Again, the statement follows because of $\gamma(\tilde{\mathbf{p}}|\mathbf{q}) = \gamma(\mathbf{p}|\mathbf{q})$ for all $\mathbf{p} \in \mathcal{P}_F$.

□

4.2 Analytical results for the transition vector $\mathbf{q} = (1, 0, 0; q, 0, 0)$

In the following, we describe in more detail the analytical results obtained for the single-stochastic game with transition vector $\mathbf{q} = (1, 0, 0; q, 0, 0)$. First, we again consider the case of weak selection, $\beta \to 0$. By Proposition 2, we know that without information, the game leads to a long-run average cooperation rate of $\hat{\gamma}_0^N(\mathbf{q}) = 1/2$. In the following, we discuss the case of full information. To this end, we note that for the given transition vector $\mathbf{q}$, the space of full-information memory-1 strategies is 5-dimensional, consisting of vectors $\mathbf{p} = (p^1_{CC}, p^2_{CC}, p^2_{CD}, p^2_{DC}, p^2_{DD})$, see also Supplementary Note 3.

Proposition 4. *When players have full information on the current state, the long-run cooperation rate of the population in the limit of rare errors is*

$$\hat{\gamma}_0^F = 1/2 - \frac{3q(1-q)}{64(1+q)}. \tag{79}$$

In particular, $\hat{\gamma}_0^F \le 1/2$*, with equality if and only if* $q=0$ *or* $q=1$*. Moreover, the function* $\hat{\gamma}_0^F$ *is convex for* $0 \le q \le 1$ *and has a unique minimum at* $q^* = \sqrt{2}-1 \approx 0.41$*.*

Proof. The proof is by explicitly computing the cooperation rate $\gamma(\mathbf{p}|\mathbf{q})$ for all 32 possible resident strategies. As an example, when both players adopt the resident strategy $\mathbf{p}=(0,1,0,0,1)$ in the stochastic game with transition vector $\mathbf{q}$, the Markov chain according to Eq. (55) takes the following form,

$$M(\mathbf{p}|\mathbf{q}) = \begin{pmatrix} 0 & 0 & 0 & 1 & 0 & 0 & 0 & 0 \\ 0 & 0 & 0 & 0 & 0 & 0 & 0 & 1 \\ 0 & 0 & 0 & 0 & 0 & 0 & 0 & 1 \\ 0 & 0 & 0 & 0 & 1 & 0 & 0 & 0 \\ 0 & 0 & 0 & q & 1-q & 0 & 0 & 0 \\ 0 & 0 & 0 & 0 & 0 & 0 & 0 & 1 \\ 0 & 0 & 0 & 0 & 0 & 0 & 0 & 1 \\ 0 & 0 & 0 & 0 & 1 & 0 & 0 & 0 \end{pmatrix}. \tag{80}$$

The respective invariant distribution is

$$\mathbf{v}(\mathbf{p}|\mathbf{q}) = \left(0,0,0,\frac{q}{1+q},\frac{1}{1+q},0,0,0\right). \tag{81}$$

It follows that the resulting average cooperation rate is

$$\gamma(\mathbf{p}|\mathbf{q}) = \frac{1}{1+q}. \tag{82}$$

By repeating the same computation for all other strategies $\mathbf{p}=(p^1_{CC}, p^2_{CC}, p^2_{CD}, p^2_{DC}, p^2_{DD})$, we obtain

$$
\begin{array}{llll}
\gamma\big((0,0,0,0,0)\big|\mathbf{q}\big)=0, & \gamma\big((0,1,0,0,0)\big|\mathbf{q}\big)=0, & \gamma\big((1,0,0,0,0)\big|\mathbf{q}\big)=0, & \gamma\big((1,1,0,0,0)\big|\mathbf{q}\big)=0,\\
\gamma\big((0,0,0,0,1)\big|\mathbf{q}\big)=\frac{1}{2}, & \gamma\big((0,1,0,0,1)\big|\mathbf{q}\big)=\frac{1}{1+q}, & \gamma\big((1,0,0,0,1)\big|\mathbf{q}\big)=1, & \gamma\big((1,1,0,0,1)\big|\mathbf{q}\big)=1,\\
\gamma\big((0,0,0,1,0)\big|\mathbf{q}\big)=\frac{1}{4}, & \gamma\big((0,1,0,1,0)\big|\mathbf{q}\big)=\frac{1}{4}, & \gamma\big((1,0,0,1,0)\big|\mathbf{q}\big)=\frac{1+q}{4}, & \gamma\big((1,1,0,1,0)\big|\mathbf{q}\big)=\frac{1}{2},\\
\gamma\big((0,0,0,1,1)\big|\mathbf{q}\big)=\frac{1}{2}, & \gamma\big((0,1,0,1,1)\big|\mathbf{q}\big)=\frac{3+q}{4+4q}, & \gamma\big((1,0,0,1,1)\big|\mathbf{q}\big)=\frac{3}{4}, & \gamma\big((1,1,0,1,1)\big|\mathbf{q}\big)=\frac{3}{4},\\
\gamma\big((0,0,1,0,0)\big|\mathbf{q}\big)=\frac{1}{4}, & \gamma\big((0,1,1,0,0)\big|\mathbf{q}\big)=\frac{1}{4}, & \gamma\big((1,0,1,0,0)\big|\mathbf{q}\big)=\frac{1+q}{4}, & \gamma\big((1,1,1,0,0)\big|\mathbf{q}\big)=\frac{1}{2},\\
\gamma\big((0,0,1,0,1)\big|\mathbf{q}\big)=\frac{1}{2}, & \gamma\big((0,1,1,0,1)\big|\mathbf{q}\big)=\frac{3+q}{4+4q}, & \gamma\big((1,0,1,0,1)\big|\mathbf{q}\big)=\frac{3}{4}, & \gamma\big((1,1,1,0,1)\big|\mathbf{q}\big)=\frac{3}{4},\\
\gamma\big((0,0,1,1,0)\big|\mathbf{q}\big)=0, & \gamma\big((0,1,1,1,0)\big|\mathbf{q}\big)=0, & \gamma\big((1,0,1,1,0)\big|\mathbf{q}\big)=q, & \gamma\big((1,1,1,1,0)\big|\mathbf{q}\big)=1,\\
\gamma\big((0,0,1,1,1)\big|\mathbf{q}\big)=\frac{1}{2} & \gamma\big((0,1,1,1,1)\big|\mathbf{q}\big)=\frac{1}{1+q} & \gamma\big((1,0,1,1,1)\big|\mathbf{q}\big)=1, & \gamma\big((1,1,1,1,1)\|\mathbf{q}\big)=1.
\end{array}
$$

To obtain the population's long-run average cooperation rate in the limit of rare mutations and vanishing selection, we compute the average of these 32 cooperation probabilities. This yields formula (79). □

Based on our previous results, we can also draw some conclusions for positive selection strengths. First, we note that for $q=0$, the transition vector takes the form $\mathbf{q} = (1,0,0;0,0,0)$. This transition vector has an absorbing state (state 2). Therefore, it follows from the first case in Proposition 3 that $V_\beta(\mathbf{q})=0$ for all selection strengths $\beta \geq 0$.

Second, we note that a similar conclusion holds for $q = 1$. In that case, the transition vector takes the form $\mathbf{q} = (1,0,0;1,0,0)$, and hence it is deterministic and state-independent. By the fourth case in Proposition 3 it again follows that $V_\beta(\mathbf{q})=0$ for all $\beta \geq 0$.

Finally, for a fixed $q \in (0,1)$, Proposition 4 implies that $V_0(\mathbf{q}) < 0$. Because the map $\beta \mapsto V_\beta(\mathbf{q})$ is continuous in β, it follows that we can find some $\beta^*_q > 0$ such that for all $\beta < \beta^*_q$ we have $V_\beta(\mathbf{q}) < 0$. That is, for any q strictly between 0 and 1, we do not only observe a benefit of ignorance for no selection; there is also a benefit of ignorance for weak but positive selection (see also Fig. 5**c**). However, as our numerical computations suggest, this benefit of ignorance may turn turn into a benefit of information for sufficiently large q and appropriate selection strengths β (Fig. 5d-f).

Supplementary References

[1] Wang, G., Su, Q. & Wang, L. Evolution of state-dependent strategies in stochastic games. Journal of Theoretical Biology 110818 (2021).

[2] Blackwell, D. Discounted dynamic programming. The Annals of Mathematical Statistics **36**, 226–235 (1965).

[3] Nowak, M. & Sigmund, K. A strategy of win-stay, lose-shift that outperforms tit-for-tat in the prisoner's dilemma game. Nature **364**, 56–58 (1993).

[4] Traulsen, A., Pacheco, J. M. & Nowak, M. A. Pairwise comparison and selection temperature in evolutionary game dynamics. Journal of theoretical biology **246**, 522–529 (2007).

[5] Couto, M. C., Giaimo, S. & Hilbe, C. Introspection dynamics: A simple model of counterfactual learning in asymmetric games. New Journal of Physics **24**, 063010 (2022).

Supplementary Figures

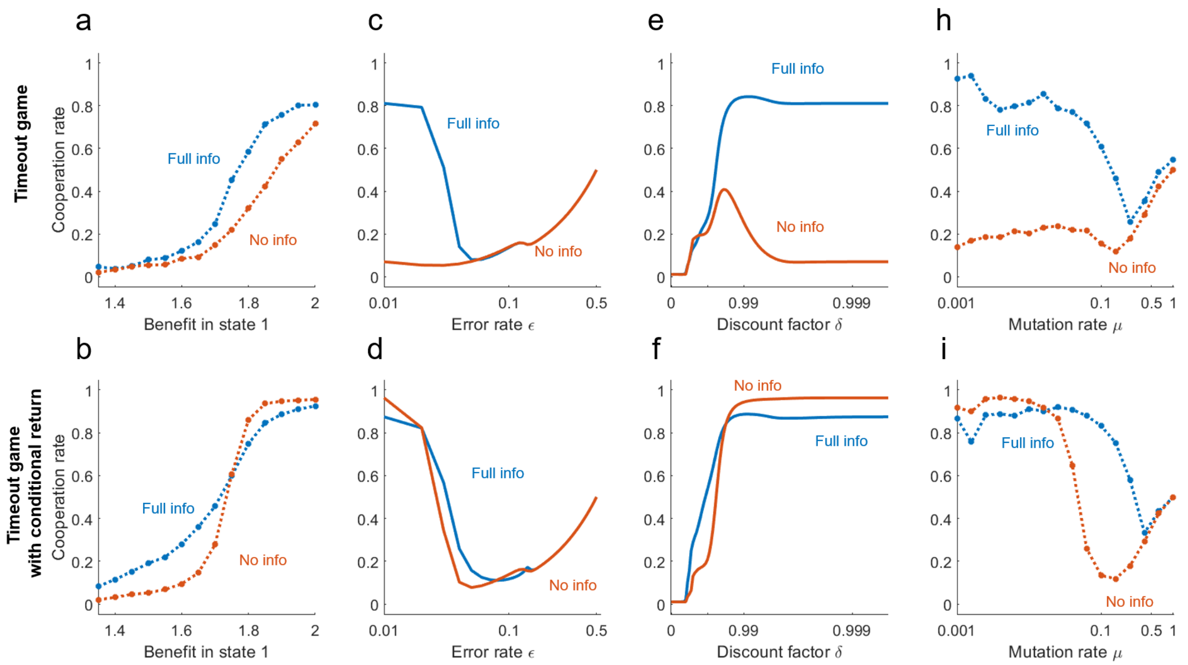

Figure S1: Robustness of our results with respect to parameter changes. To explore how different parameters affect our results, we revisit the two games introduced in Fig. 2. For both the timeout game and the timeout game with conditional return, we plot the evolving cooperation rates when there is no information (red) and full information (blue). a,b, While in the main text we have focussed on deterministic strategies, here we present simulations when players can choose among stochastic memory-1 strategies. To this end, we assume that mutating players randomly choose a new strategy whose entries are taken from an arcsine distribution, as in earlier work[3]. We find that in the game with timeout, information always provides advantage to cooperation. In the game with conditional return, ignorance is beneficial for sufficiently high values of b_1. c-i, We then independently vary the error rate ε, the discount factor δ, and the mutation rate μ. For values of parameters sufficiently close to the values in Fig. 2, we see qualitatively similar behavior. As expected, the benefit of ignorance is not robust and only observed for specific values of the parameters (d,f,i). Solid lines indicate exact numerical results in the limit of rare mutations, whereas dashed lines represent simulations. If not specified otherwise, parameter values used for these plots are the same as in Fig. 2.

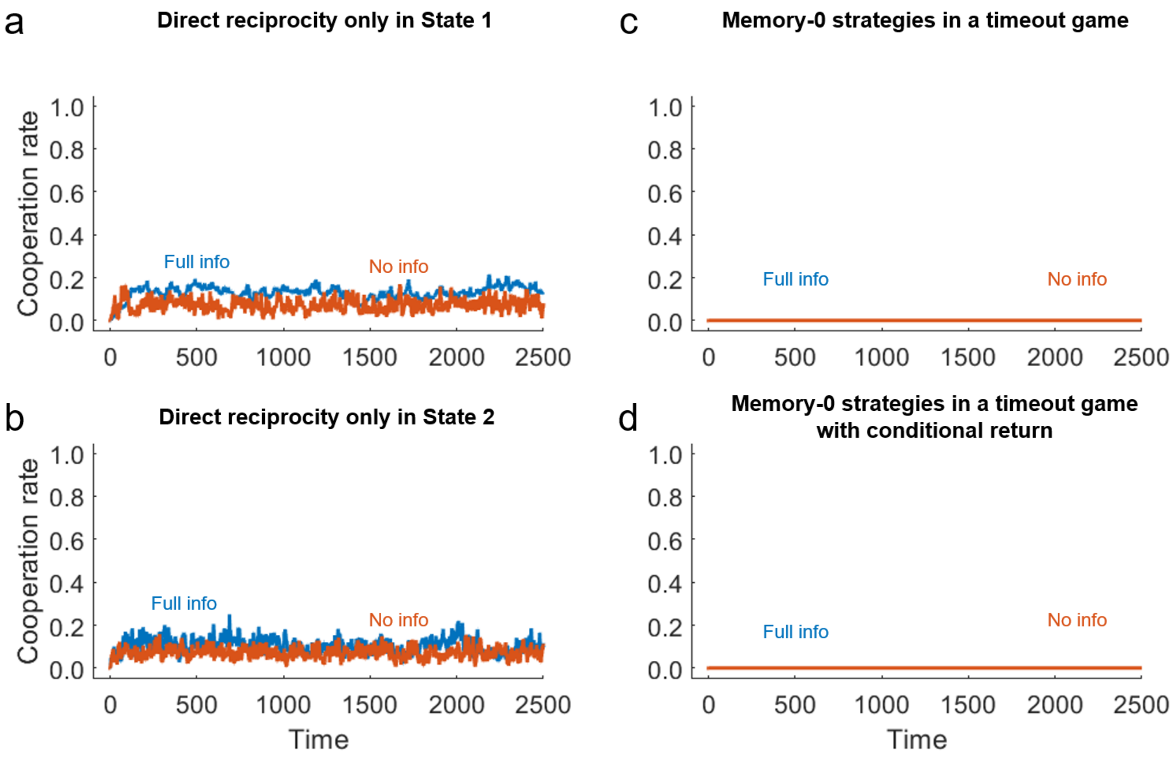

Figure S2: Robustness of our results with respect to the strategy choice. a,b, As a baseline to our results shown in Fig. 2, we explore how much cooperation evolves when players engage in a conventional repeated game (without state transitions). To this end we simulate the process with deterministic memory-1 strategies. Independent of whether we use the game in state 1 or state 2, we observe very little cooperation. We conclude that for the given parameter values, game transitions are crucial to establish cooperation. **c,d,** As another baseline, we explore whether cooperation evolves if individuals can only condition their behavior on the present state (but not on the players' actions in the last round). As one may expect, cooperation does not evolve. Parameters are the same as in Fig. 2.

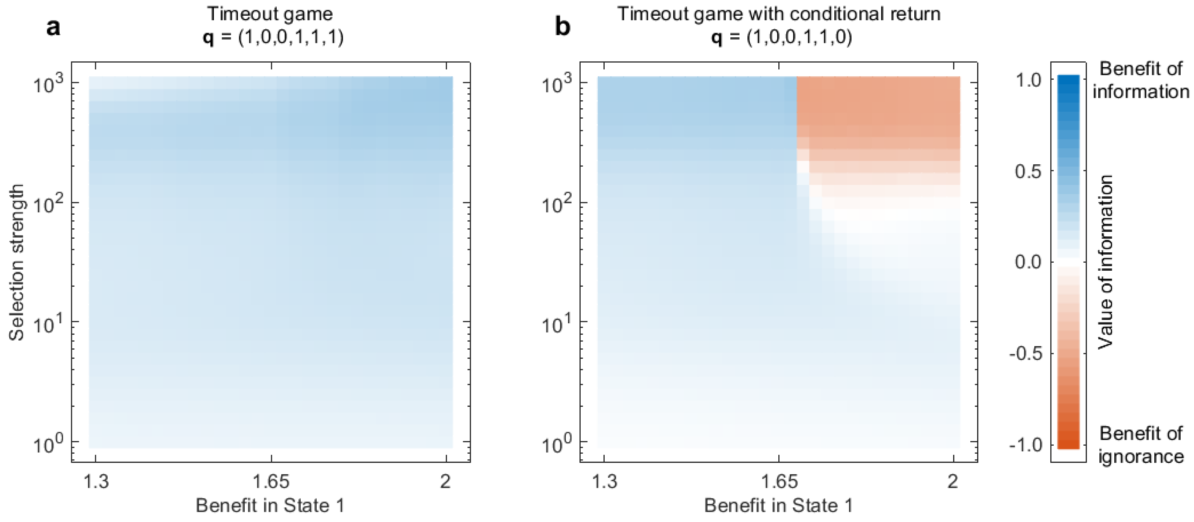

Figure S3: Value of information under an alternative learning dynamics. Our previous evolutionary results are based on an imitation dynamics, using a pairwise comparison process[4]. Here, we compare these results to a different learning process, introspection dynamics[5]. Instead of considering a population of learners, this process only involves the two two players engaged in the respective game. At regular time intervals, one player is given the chance to update its strategy. To this end, the player compares its present payoff π with the hypothetical payoff $\tilde{\pi}$ that the player could have obtained by using a randomly determined alternative strategy. The player then switches to the alternative strategy with probability $(1+\exp[-\beta(\tilde{\pi}-\pi)])^{-1}$. Here, $\beta \geq 0$ is again a parameter referred to as selection strength. For this figure, we have simulated this elementary updating process for 10^7 time steps, for the two games depicted in Fig. 2. To this end, again we assume that players can choose among all deterministic memory-1 strategies either with full information or with no information. In general, introspection dynamics is quite different from pairwise comparison[5] (in particular, introspection dynamics requires larger values of β to approximate strong selection). As a consequence, the two processes lead to different absolute cooperation rates. However, as shown here, the two processes yield similar results on a qualitative level (compared to Fig. 2d,h). **a,** In the timeout game, we observe a benefit of information for all considered parameter values. **b,** In the game with conditional return, large benefits b_1 and large selection strengths β lead to a benefit of ignorance. Unless noted otherwise, we use the same parameters as in Fig. 2.

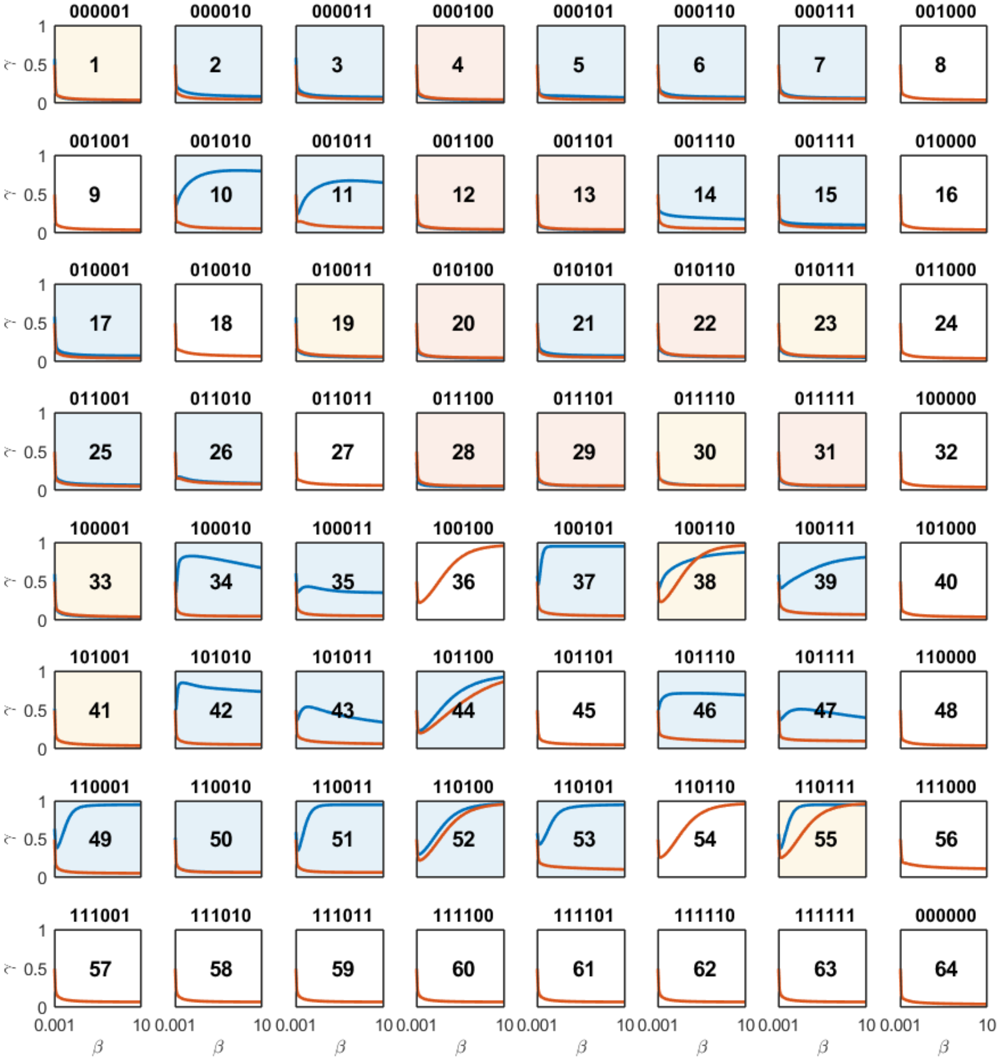

Figure S4: An analysis of all deterministic transition structures for different selection strengths. We plot cooperation rates for the games with full information (blue line) and no information (red line) as a function of the selection strength β. For each of the $2^6 = 64$ deterministic transition structures, we use colors to indicate the qualitative behavior. Cases with a consistent benefit of information are colored blue, whereas cases with a consistent benefit of ignorance are colored red. Yellow panels indicate that we observe both a benefit of information and of ignorance for different values of β. Finally, in the white panels, information is neutral. Unless noted otherwise, parameters are the same as in Fig. 2.

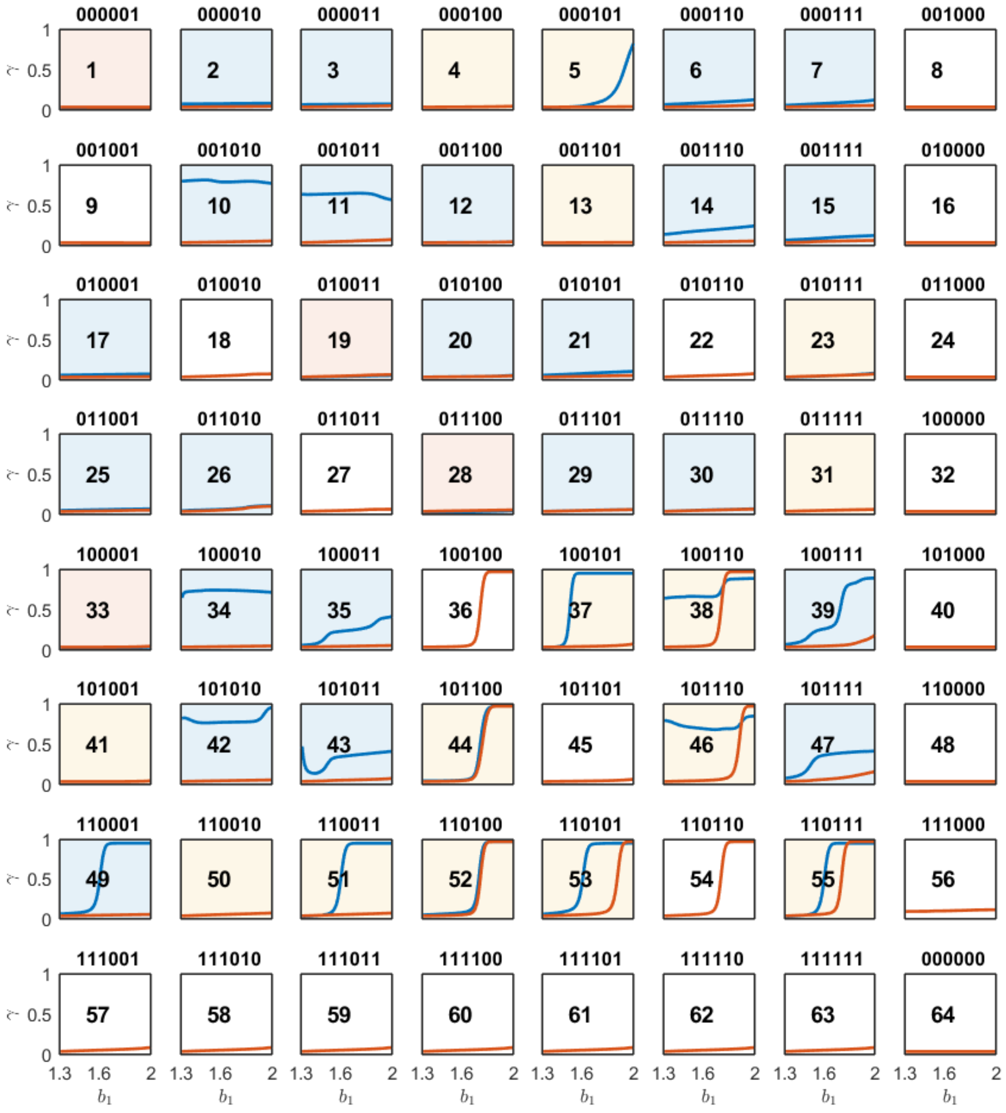

Figure S5: An analysis of all deterministic transition structures for different benefits in game 1. Again, we plot cooperation rates for games with full information (blue line) and no information (red line), but this time as a function of b_1. For the panels, we use the same color code as before (blue – benefit of information, red – benefit of ignorance, yellow – inconsistent, white – neutral). Unless noted otherwise, parameters are the same as in Fig. 2.

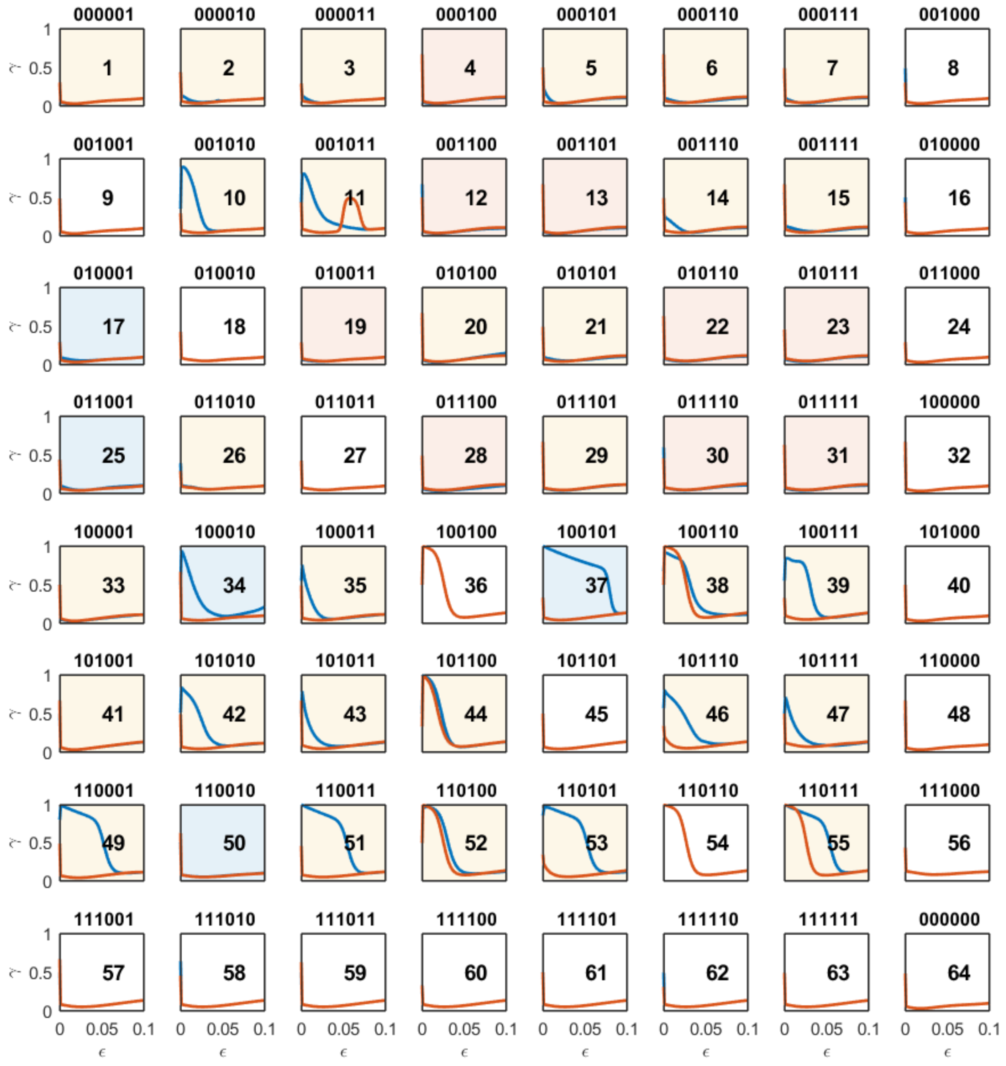

Figure S6: An analysis of all deterministic transition structures for different error rates. As before, we plot cooperation rates for games with full information (blue line) and no information (red line), now as a function of the error rate ε. The panels use the same color code as before (blue – benefit of information, red – benefit of ignorance, yellow – inconsistent, white – neutral). Unless noted otherwise, parameters are the same as in Fig. 2.

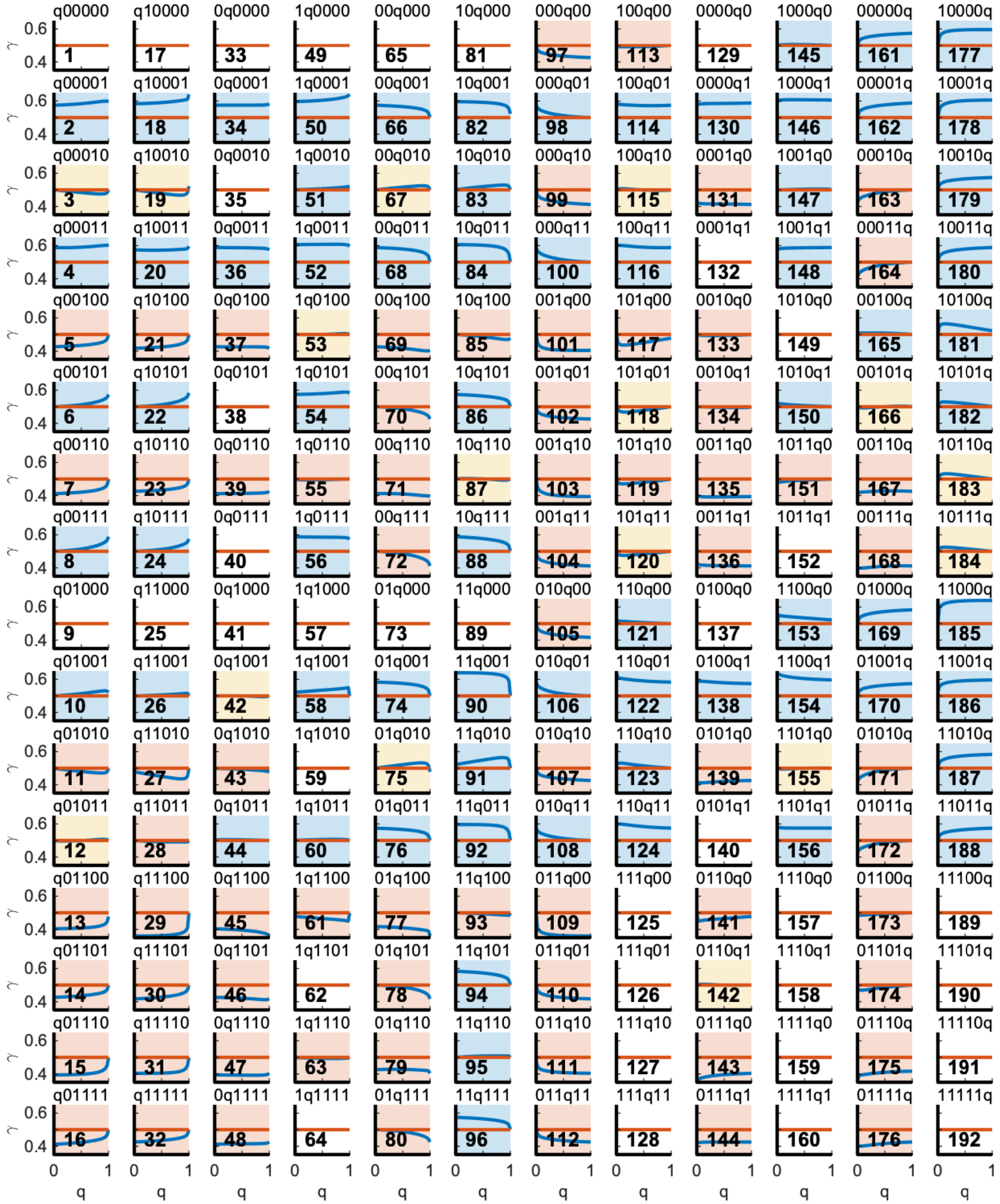

Figure S7: An analysis of all single-stochastic games for vanishing selection. As the most simple instantiation of games with an element of chance, we consider all $6\cdot2^5=192$ families of single-stochastic games. Here, exactly one transition is probabilistic (and we use q as the respective transition probability). For each family, we reproduce the cooperation rates for full information (blue curve) and no information (red curve). No information always yields 50% cooperation. Full information can yield the same cooperation rate (white), more cooperation (blue) or less cooperation (red). We use the same baseline parameters as in Fig. 2, but with a selection strength $\beta=0$.

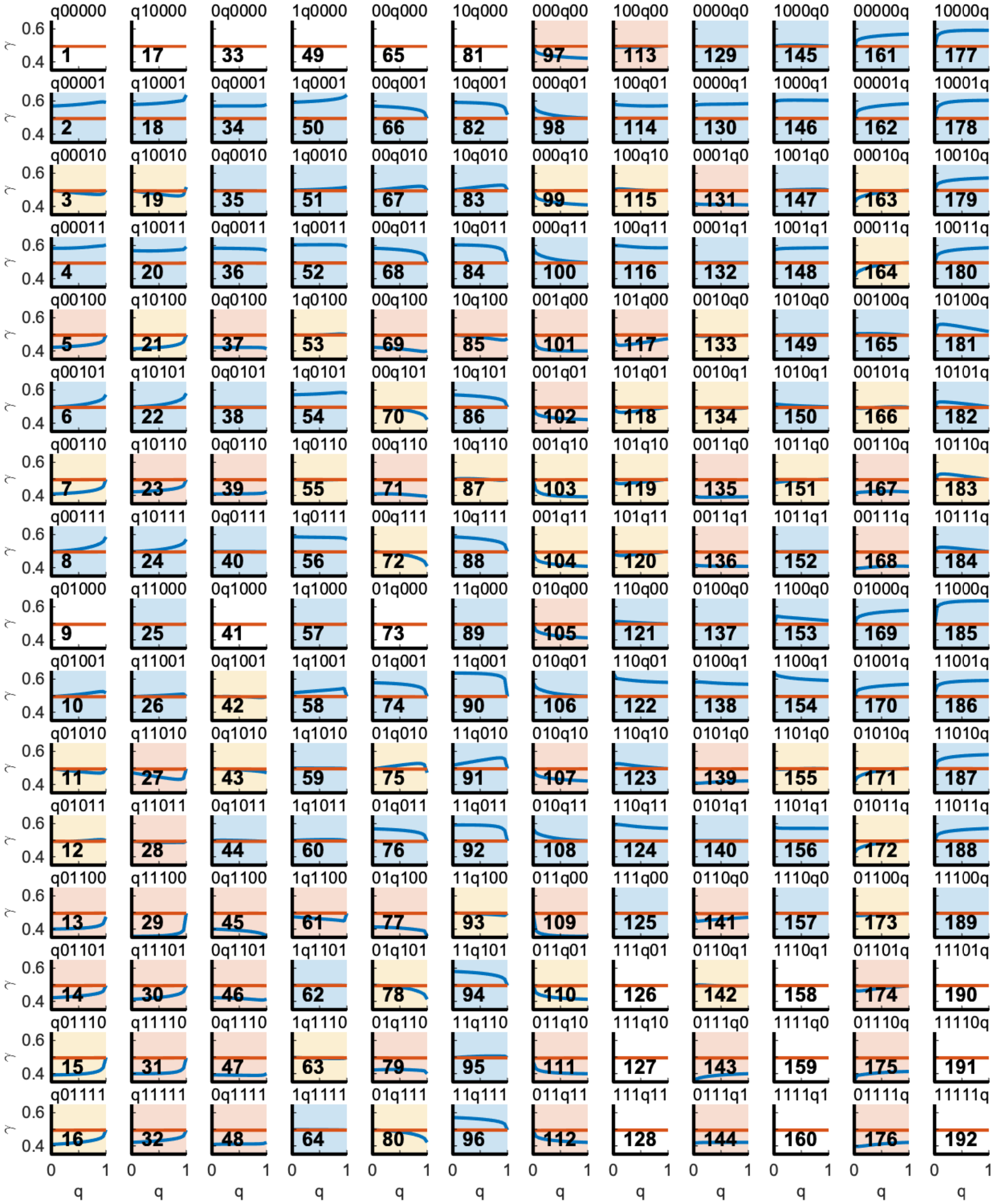

Figure S8: An analysis of all single-stochastic games for weak selection. Same as Fig. S7, but for $\beta = 0.001$ instead of $\beta = 0$.

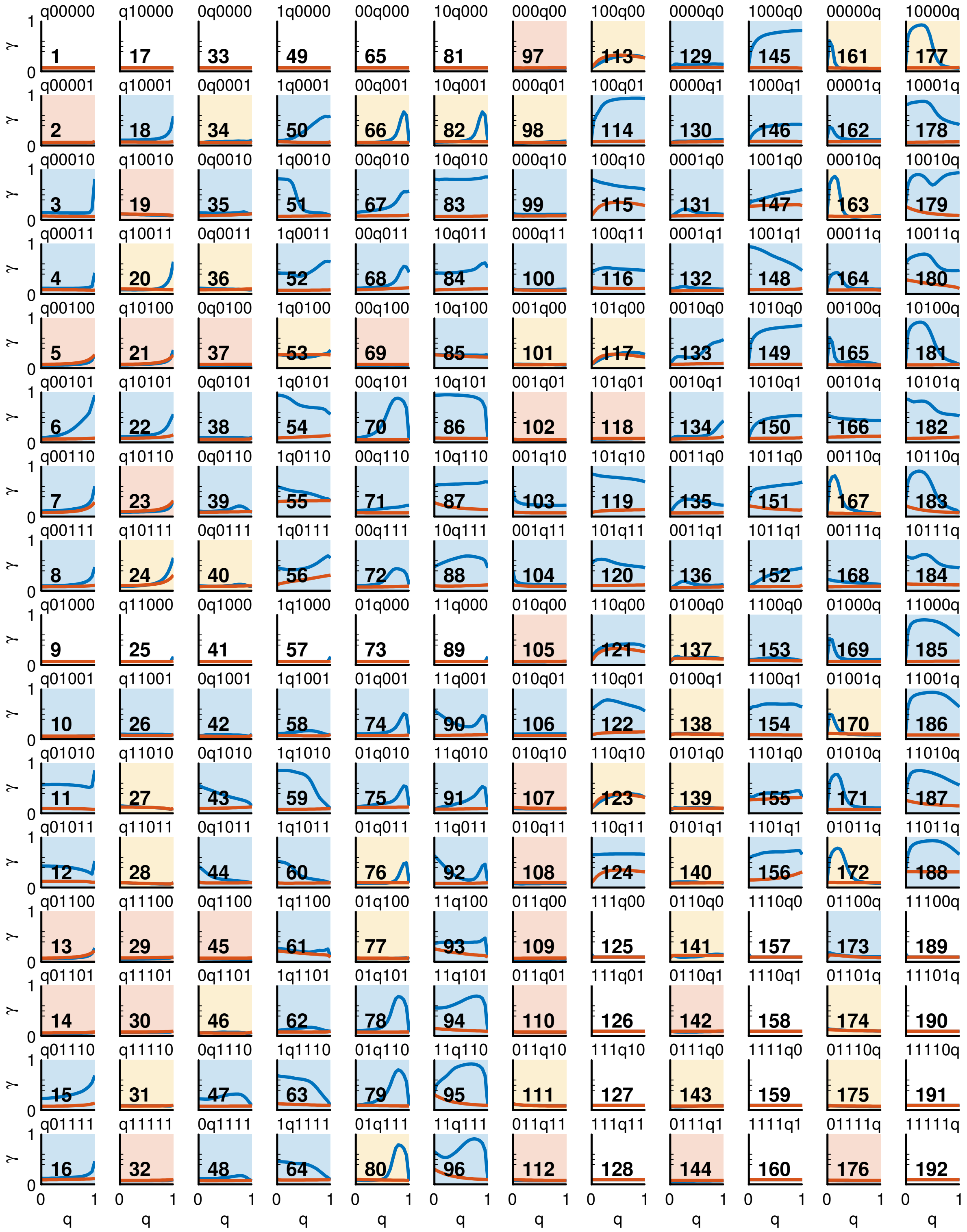

Figure S9: An analysis of all single-stochastic games for intermediate selection. Same as previous two figures, but using $\beta = 1$.

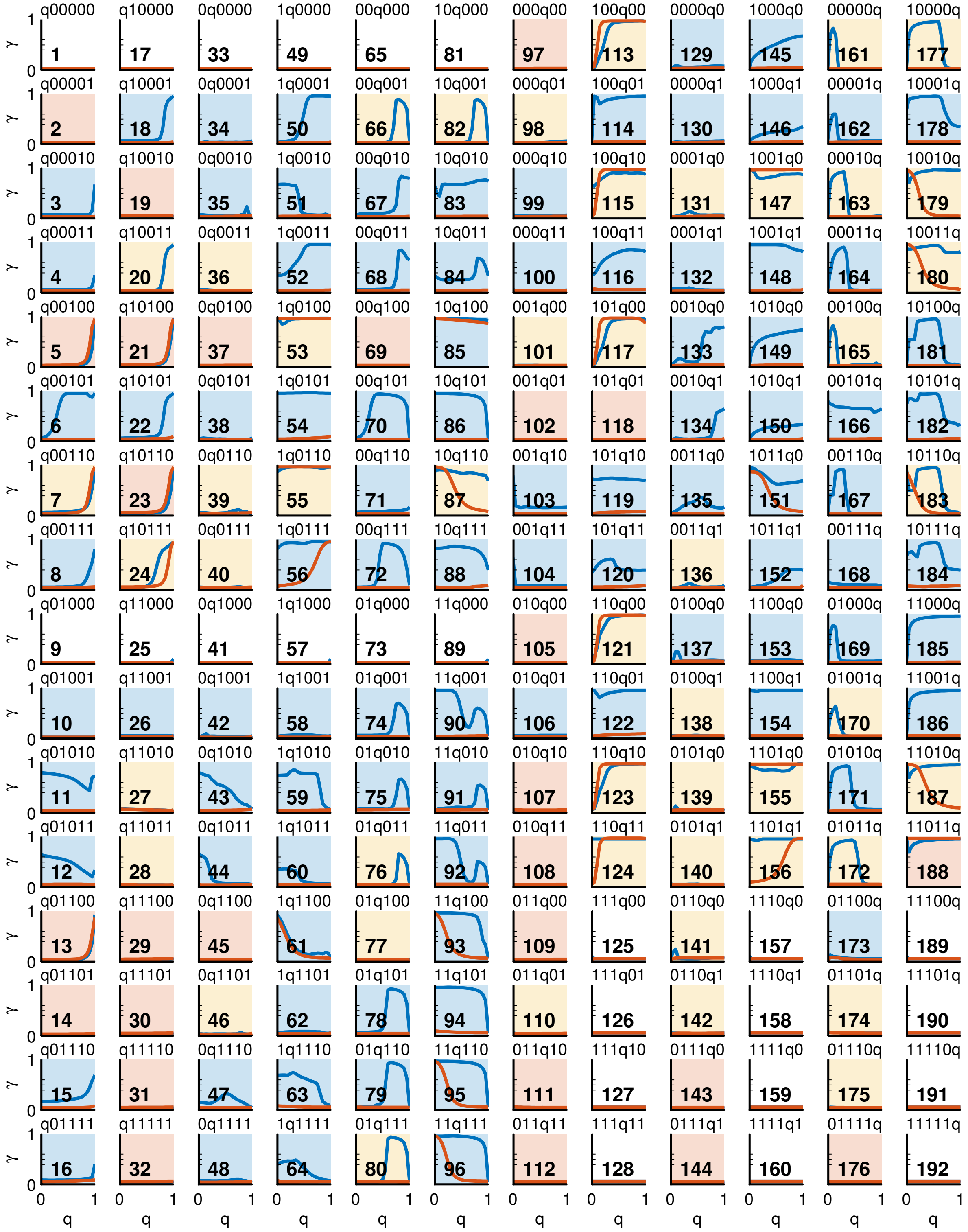

Figure S10: An analysis of all single-stochastic games for strong selection. Same as previous figures, but with $\beta = 10$.

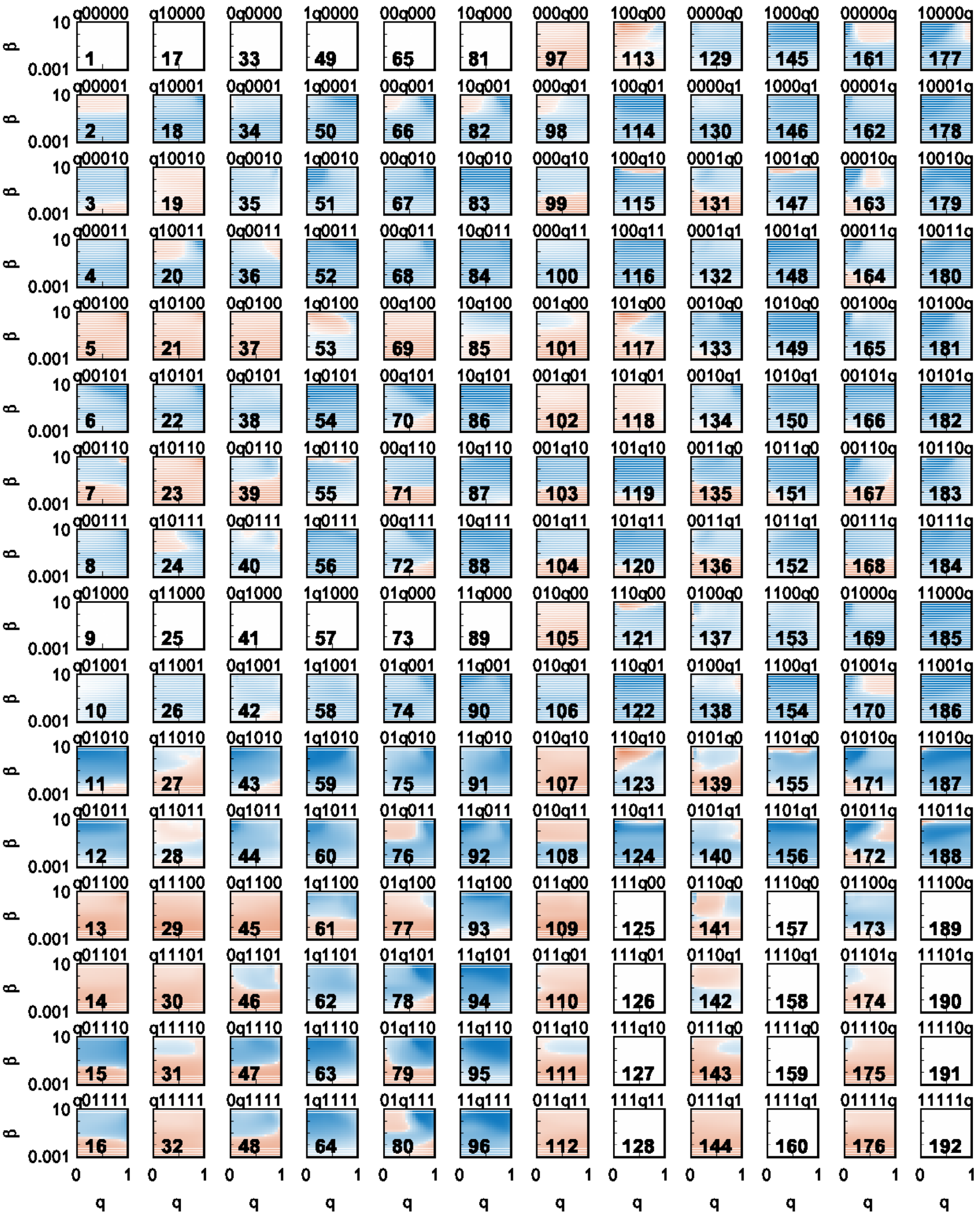

Figure S11: A systematic analysis of the single-stochastic transition structures for positive selection strength. The figure reproduces the contour plot of Fig. 5 for all 192 single-stochastic games. There are exactly 24 transition structures for which there is no difference between full and no information for any selection strength. In all these games, one of the two environmental states is absorbing. Parameter values are the same as in Fig. 5.

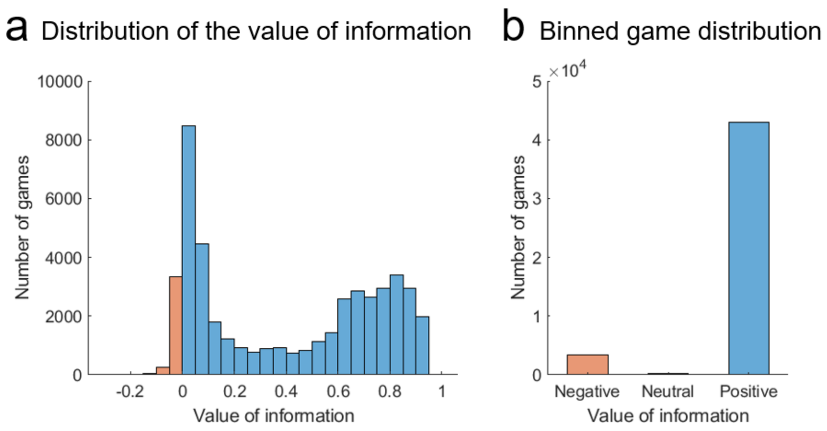

Figure S12: Value of information in games with stochastic transitions. a, We assume the entries of $\mathbf{q}$ are taken from a finite grid $q_{ij}^{k} \in \{0, 0.2, 0.4, 0.6, 0.8, 1.0\}$, giving rise to $6^6 = 46,656$ different games. For each game, we calculate the average cooperation rates for full and no information. We plot the distribution of the resulting value of information for all considered stochastic games. **b,** We bin the games into three categories: games with positive (blue), negative (red) and neutral (white) value of information. Most games exhibit a benefit of information. Parameter values are the same as in Fig. 2.

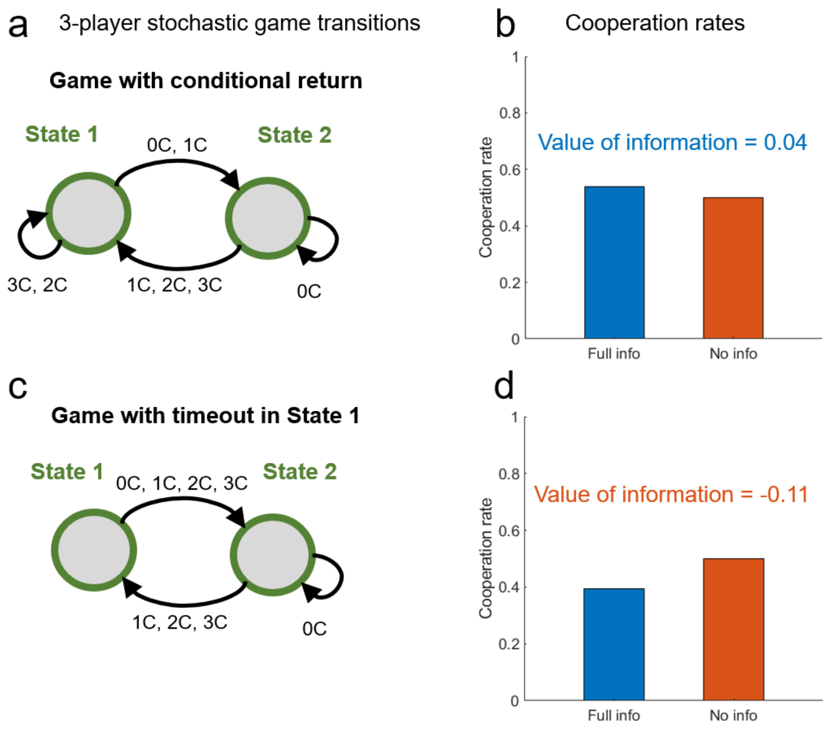

Figure S13: Effect of information in two 3-player games. a,b, We analyze a three-player public good game where players move for one round to the worse state 2 if at least two players defect. We compute cooperation rates for full information and no information, in the limit of no selection $\beta = 0$. We find a benefit of information. **c,d,** We perform the same analysis for a different 3-player game. Here, we find a benefit of ignorance. Parameter values: $r_1 = 1.6$, $r_2 = 1$, $c = 1$, population size $N = 100$, error rate $\varepsilon = 0.01$.
